# Update of the recommendations of the *Sociedade Portuguesa de Cuidados Intensivos* and the Infection and Sepsis Group for the approach to COVID-19 in Intensive Care Medicine

**DOI:** 10.5935/0103-507X.0103-507X-rbti-20210080

**Published:** 2021

**Authors:** João João Mendes, José Artur Paiva, Filipe Gonzalez, Paulo Mergulhão, Filipe Froes, Roberto Roncon, João Gouveia

**Affiliations:** 1 Sociedade Portuguesa de Cuidados Intensivos - Lisboa, Portugal.; 2 Department of Intensive Care Medicine, Hospital Prof. Doutor Fernando da Fonseca EPE - Lisboa, Portugal.; 3 College of Specialties of Intensive Care Medicine, Ordem dos Médicos- Lisboa, Portugal.; 4 Infection and Sepsis Group - Lisboa, Portugal.; 5 Department of Intensive Care Medicine, Centro Hospitalar Universitário de São João EPE, Faculdade de Medicina da Universidade do Porto - Porto, Portugal.; 6 Department of Intensive Care Medicine, Hospital Garcia de Orta EPE - Lisboa, Portugal.; 7 Polyvalent Intensive Care Unit, Hospital Lusíadas Porto - Porto, Portugal.; 8 Medical-Surgical Intensive Care Unit, Hospital de Pulido Valente, Centro Hospitalar Universitário de Lisboa Norte EPE - Lisboa, Portugal.; 9 Department of Intensive Care Medicine, Centro Hospitalar Universitário de Lisboa Norte EPE - Lisboa, Portugal.

**Keywords:** COVID-19/therapy, COVID-19/diagnosis, Coronavirus infections, SARS-CoV-2, Practice guidelines as topic

## Abstract

**Introduction:**

The *Sociedade Portuguesa de Cuidados Intensivos* and the Infection and Sepsis Group have previously issued health service and management recommendations for critically ill patients with COVID-19. Due to the evolution of knowledge, the panel of experts was again convened to review the current evidence and issue updated recommendations.

**Methods:**

A national panel of experts who declared that they had no conflicts of interest regarding the development of the recommendations was assembled. Operational questions were developed based on the PICO methodology, and a rapid systematic review was conducted by consulting different bibliographic sources. The panel determined the direction and strength of the recommendations using two Delphi rounds, conducted in accordance with the principles of the GRADE system. A strong recommendation received the wording “is recommended”, and a weak recommendation was written as “is suggested.”

**Results:**

A total of 48 recommendations and 30 suggestions were issued, covering the following topics: diagnosis of SARS-CoV-2 infection, coinfection and superinfection; criteria for admission, cure and suspension of isolation; organization of services; personal protective equipment; and respiratory support and other specific therapies (antivirals, immunomodulators and anticoagulation).

**Conclusion:**

These recommendations, specifically oriented to the Portuguese reality but that may also apply to Portuguese-speaking African countries and East Timor, aim to support health professionals in the management of critically ill patients with COVID-19. They will be continuously reviewed to reflect the progress of our understanding and the treatment of this pathology.

## INTRODUCTION

Coronavirus 2019 (COVID-19), the disease caused by severe acute respiratory syndrome coronavirus 2 (SARS-CoV-2), was initially reported in the city of Wuhan, in the Chinese province of Hubei, and rapidly spread throughout China, with subsequent involvement of multiple countries. The World Health Organization (WHO) classified the COVID-19 epidemic as a pandemic on March 11, 2021,^(^1^)^ when the disease had already been identified in 114 countries. In Portugal, the first cases were reported on March 2, 2021, and the first death from the disease occurred on March 16, 2021.^(^2^)^

In response to the COVID-19 pandemic, *Sociedade Portuguesa de Cuidados Intensivos* and the Infection and Sepsis Group issued recommendations aimed at organizing intensive care services as well as providing diagnosis and treatment (supportive and specific) guidance for critically ill patients with COVID-19.^(^3^)^ Due to the evolution of knowledge, the panel of experts was again convened to review the current evidence and issue updated recommendations.

The present document is divided into two sections: (1) a review of SARS-CoV-2 virology and the clinical presentation of COVID-19 and (2) health services and management recommendations/suggestions for patients with COVID-19 in intensive care departments.

## METHODS

A national panel of experts was invited by the heads of the *Sociedade Portuguesa de Cuidados Intensivos* and the Infection and Sepsis Group to prepare these recommendations. All panel members declared that they had no conflicts of interest regarding the development of the recommendations.

The first iteration of the Recommendations of the *Sociedade Portuguesa de Cuidados Intensivos* and the Infection and Sepsis Group for the approach to COVID-19 in Intensive Care Medicine was used as the basis^(^3^)^ for the adaptation and development of these new recommendations. Communication and elaboration among the group were facilitated by electronic mail and teleconference, with the online sharing of a central document. Clinical questions were asked, emphasizing measures of potential impact on the organization of health services and the management of patients with COVID-19 in intensive care departments. For each question, operational questions were developed in accordance with the PICO methodology (participants, interventions, comparisons and outcomes),^(^4^)^ with the population of interest being patients with COVID-19 requiring hospitalization in intensive care departments. For each clinical question, a rapid systematic review was conducted by reviewing the topics listed in the DynaMed and UptoDate databases; several bibliographic searches, with an emphasis on systematic reviews and clinical trials, in PubMed® and in the Cochrane Library used different combinations of search terms related to infection by SARS-CoV-2 and COVID-19 throughout the period encompassing the preparation of the document and review of the topics related to the norms and normative circulars of the Directorate-General for Health (DGS; available at https://www.dgs.pt/normas-orientacoes-e-informacoes/normas-e-circulares-normativas.aspx)

Finally, the panel determined the direction (positive or negative) and strength (strong or weak) of the recommendations using two Delphi rounds (self-administered questionnaire, without meetings between the participants).^(^5^)^ All these procedures were conducted in accordance with the principles of the GRADE system and taking into account the following factors: quality of evidence, certainty of the balance between advantages and disadvantages, certainty or similarity in values and preferences, and implications of resources. To obtain a consensus, an average level of agreement equal to or greater than 80% was required. When the level of agreement was less than 80%, additional discussion and voting were conducted. A strong recommendation received the wording “is recommended,” and a weak recommendation was written as “is suggested.”

## SARS-CoV-2

### Virological characteristics

SARS-CoV-2 is a simple positive-sense RNA (ribonucleic acid) genome virus belonging to the genus Betacoronaviruses (β-CoV), whose virion has four structural proteins. The structural proteins S (*spike*), E (envelope) and M (membrane) create the viral envelope, and protein N (nucleus) contains the RNA genome.

There are four strains (HCoV-229E, HCoV-NL63, HCoV-OC43 and HCoV-HKU1) that circulate seasonally in the human population, most often causing low-severity respiratory infections (for example, constipation) and, rarely, viral pneumonia.^(^6^)^ Until the emergence of SARS-CoV-2,^(^7^)^ two other strains that caused epidemic outbreaks with zoonotic origin had been described that passed the species barrier. One of the strains is severe acute respiratory syndrome coronavirus (SARS-CoV-1), which originated from bats and was transmitted to the African civet; SARS-CoV-1 causes severe acute respiratory syndrome (SARS) in humans and circulated between 2002 and 2004. The other strain is Middle East respiratory syndrome coronavirus (MERS-CoV), which originated from bats and was transmitted to camelids; MERS-CoV causes Middle East respiratory syndrome (MERS) and has circulated since 2012.^(^8^)^ Both SARS-CoV-1 and MERS-CoV have a high mortality rate and may present as acute respiratory failure, requiring invasive mechanical ventilation, vasopressor support and renal replacement techniques.^(^8^)^ SARS-CoV-2 shares a genetic identity of 96.2% with a coronavirus circulating in natural populations of bats of the species *Rhinolophus affinis* (SARSr-Ra-Bat-CoV-RaTG13.9), and the pangolin has been hypothesized as the intermediate host.^(^6^)^

The basic number of reproductions (R0, number of new cases generated from a single confirmed case in a completely susceptible population) is an indicator of the transmissibility of infection and should be calculated in the initial phase of an epidemic (without considering the implementation of containment and delay measures). Based on the epidemic curve until March 16, 2020, in Portugal, the R0 was estimated at 2.02, with a 95% confidence interval of 1.92 to 2.11.^(^9^)^ The effective reproduction number (R(t)) represents the potential effective propagation of a virus under certain conditions as a function of time and is influenced by public health interventions.^(^10^)^

SARS-CoV-2, similar to what occurs with other viruses (especially RNA), undergoes frequent changes or mutations. The mutations detected thus far have not changed the biological properties of SARS-CoV-2 responsible for the characteristics of the disease, and the new viruses are considered variants of SARS-CoV-2, not new strains. The Centers for Disease Control and Prevention (CDC) classifies the SARS-CoV-2 variants into variants of interest (which have specific genetic markers but still no clinical/epidemiological evidence), variants of concern (for which there is clinical/epidemiological evidence of increased transmissibility, more severe disease, an increase in hospitalizations or deaths, a significant reduction in neutralization by antibodies generated during previous infection or vaccination, reduced efficacy of therapies/vaccines or failure in diagnostic detection) and variants with high consequences (for which the efficacy of preventive or therapeutic measures is significantly reduced in relation to the previously circulating variants).^(^11^)^ There are no variants of high consequences yet, but three variants of concern resulting from mutations in the gene encoding structural protein S have become dominant in the countries where they have been identified and spread globally, with repercussions on the epidemiology of the disease and on vaccine efficacy. These three are variant B.1.1.7 (VOC-202012/01), or the British variant, resulting from the N501Y mutation (change from asparagine - N - to tyrosine - Y - at position 501), which increases transmissibility (between 43% and 82%)^(^11^)^ and is possibly associated with higher mortality and lower effectiveness of the therapeutic use of monoclonal antibodies;^(^11^)^ variant B.1.351 (501Y.V2), initially identified in South Africa, resulting from the E484K mutation (change from glutamic acid (E) to lysine (K) at position 484), which is associated with avoidance or greater difficulty in recognition of the virus by the natural immune response^(^11^)^ or induced by a vaccine;^(^12^)^ and variant P.1 (B.1.1.28.1), identified in Manaus, Brazil, which shares the mutation (and the respective risks) of variant B.1.351.^(^11^)^ In Portugal, variant B.1.1.7. was identified in January 2021,^(^13^)^ becoming dominant in the following month; the circulation of the others was still very limited.^(^14^)^

### Forms of transmission

Effective person-to-person transmission of SARS-CoV-2 was established a few weeks after the first reported cases.^(^15^)^ The amount of virus release from the upper airways is a determinant of transmissibility, with very high viral load values in the pharynx during the first week of symptoms, with a peak around the 4th day.^(^16^)^ The viral load may be elevated 2 to 3 days before the onset of symptoms, and asymptomatic individuals may also spread the virus.^(^17^)^

Transmission occurs predominantly by inhalation - and possibly by contact with mucous membranes (for example, ocular and digestive) - of respiratory droplets (macro droplets, which are particles with a diameter > 5mm that, because of the effect of gravity, travel distances less than 1m) expelled in the course of interactions between people in close contact (usually less than 1m).^(^18^)^ The virus persists on inanimate surfaces for up to 72 hours,^(^19^)^ but there are no convincing data supporting transmission through fomites (inanimate objects or substances capable of absorbing, retaining and transporting infectious agents) or surfaces of common use.^(^20^)^ Airborne transmission via aerosols (microdroplets, particle diameter > 5µm) in the community seems to be more of an exception than the rule.^(^21^)^ However, in the hospital setting (particularly in intensive care departments), airborne transmission should always be considered during the provision of potentially aerosol-generating clinical care (for example, intubation, aspiration of secretions and bronchoscopy) or prolonged contact (> 15 minutes) and/or intimate contact (e.g., placement of a central venous catheter, surgery and cardiorespiratory resuscitation maneuvers).^(^22^)^

SARS-CoV-2 transmission can potentially occur through other pathways, such as fecal-oral transmission, because the presence of viral genetic material has been detected in the feces (but not in the urine) of patients, maintained for periods longer than in respiratory samples,^(^23^)^ and parenteral transmission, although the presence of viral genetic material has been rarely detected in blood products.^(^24^)^ Although the probability of transmission through these last two pathways has not been established, the handling of feces and blood from confirmed cases must be conducted in accordance with strict safety measures.^(^24^)^

## COVID-19

### Pathophysiology

**Figure 1:**
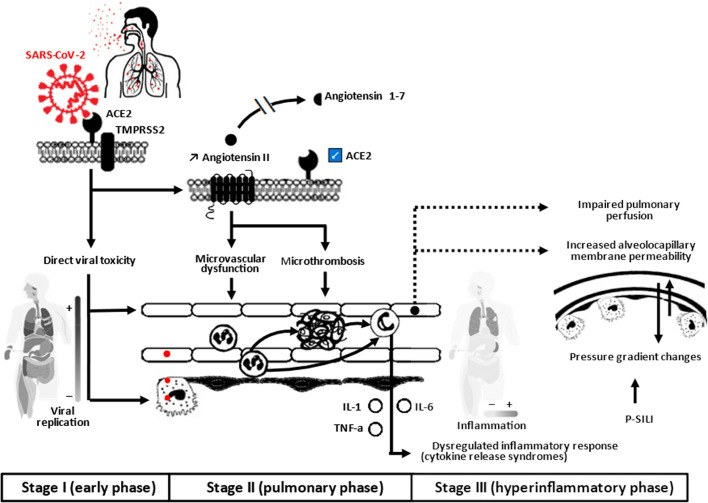
Pathophysiology of COVID-19.

The pathophysiological processes associated with COVID-19 are summarized in figure 1 (Appendix 1). SARS-CoV-2 infection occurs after contact with a significant inoculum, and viral uptake occurs in target cells that coexpress angiotensin-converting enzyme 2 (ACE2) and transmembrane serine protease 2 (TMPRSS2). SARS-CoV-2 interacts with the host through the structural protein S, which binds to the complementary receptor on target cells (ACE2), and TMPRSS2 is responsible for the cleavage of ACE2 and activation of S.^(^25^)^

Viral replication cycles occur initially in epithelial cells of the upper respiratory tract, with subsequent extension to segments of the lower respiratory tract, likely involving an aspiration mechanism.^(^26^)^ At the alveolar-capillary level, binding to ACE2 on epithelial cells (especially type 2 alveolar epithelial cells) and endothelial cells results in direct viral cytotoxicity and innate immune response activation but also in renin-angiotensin-aldosterone system activation via the negative regulation of ACE2 expression and the consequent reduction in the generation of angiotensin 1-7 (which has vasodilator and antiinflammatory effects) and the excessive production of angiotensin 2.^(^27^)^ This increase in angiotensin 2, associated with endothelial injury and adaptive immune response activation, results in microvascular dysfunction and microthrombosis.^(^28^)^ These local thrombo-inflammatory phenomena can be amplified, resulting in a dysregulated inflammatory response reminiscent of cytokine release syndromes,^(^29^)^ with the production of proinflammatory cytokines, particularly interleukin (IL)-1, IL-6 and tumor necrosis factor alpha (TNF-α), often associated with coagulopathy.^(^30^)^

The overlap of these pathophysiological processes translates into evolution through several stages of the disease, for example, the three-stage classification proposed by Siddiqi et al.:^(^31^)^ **stage I (early phase)**, viral replication; **stage II (pulmonary phase)**, activation of the adaptive immune response, which results in reduced viral load but initiates a thrombo-inflammatory cascade capable of causing tissue injury, with predominantly pulmonary expression; and **stage III (hyperinflammatory phase)**, dysregulated immune response, leading to cytokine storm syndrome.^(^32^)^ It is extremely important to recognize that patients do not progress through all three stages and that the diagnosis of the hyperinflammatory phase implies the exclusion of bacterial overinfection.^(^33^)^

Pulmonary manifestations are dependent on the stage of disease progression and result from the interaction of the referred pathophysiological mechanisms: dead space ventilation (due to microvascular dysfunction) and intrapulmonary shunt (due to increased permeability of the alveolar-capillary membrane and alveolar filling with inflammatory exudate). The relative preponderance of these various mechanisms, associated with possible forms of nonprotective ventilation or patient self-inflicted lung injury (P-SILI, intra-alveolar fluid transudation due to changes in the pressure gradient resulting from very negative intrathoracic pressure in the context of increased respiratory drive),^(^34^)^ probably results in different phenotypes,^(^35^,^36^)^ with distinct anatomopathological signatures.^(^37^,^38^)^

In addition to pulmonary manifestations, extrapulmonary manifestations (cardiovascular, renal, endocrinological, neurological, gastrointestinal and hepatobiliary) are also described, whose pathophysiological processes are similar to those previously described and are associated, in the early stages, with direct viral cytotoxicity (in tissues in which ACE2 and TMPRSSS2 are coexpressed, such as, myocytes, proximal renal tubular cells, podocytes, pancreatic beta cells, esophageal keratinocytes, gastrointestinal epithelial cells and cholangiocytes)^(^39^)^ and, in advanced stages, with deregulated inflammatory responses and with possible thrombo-inflammatory phenomena. Thus, for example, with regard to cardiology, myocarditis may occur (by direct cytopathic effects or inflammatory mechanisms), but the risk of acute coronary events is higher, especially in patients with previous cardiovascular disease and with higher ACE2 expression, via thrombo-inflammatory mechanisms. In the extrapulmonary environment, lymphopenia deserves special mention, occurring predominantly by direct cytotoxic action of the virus after ACE2-dependent or ACE2-independent entry,^(^40^)^ as do stomatological manifestations (namely, anosmia), which occur because nasal epithelial cells have the highest ACE2 expression in the entire respiratory tract.^(^41^)^ However, the distribution of these different pathophysiological mechanisms is not uniform^(^42^)^ because direct viral cytotoxicity, as measured by the expression of SARS-CoV-2 genetic and protein material, occurs in a wide variety of sites, including the respiratory tract and various extrapulmonary sites, and thrombo-inflammatory phenomena, measured by histological activity, are more expressive in the lung and in the reticulum endothelial system.

**Prolonged or long COVID-19**^(^43^)^ occurs 4 weeks after the initial infection and continues for a period of time not yet fully defined, presenting as multiple syndromes resulting from different pathophysiological processes along the spectrum of the disease (for example, organ dysfunction resulting from acute viral infection, a persistent hyperinflammatory state and psychological and physical weakness due to disease).

## CLINICAL

### General presentation and pulmonary manifestations

**Figure 2:**
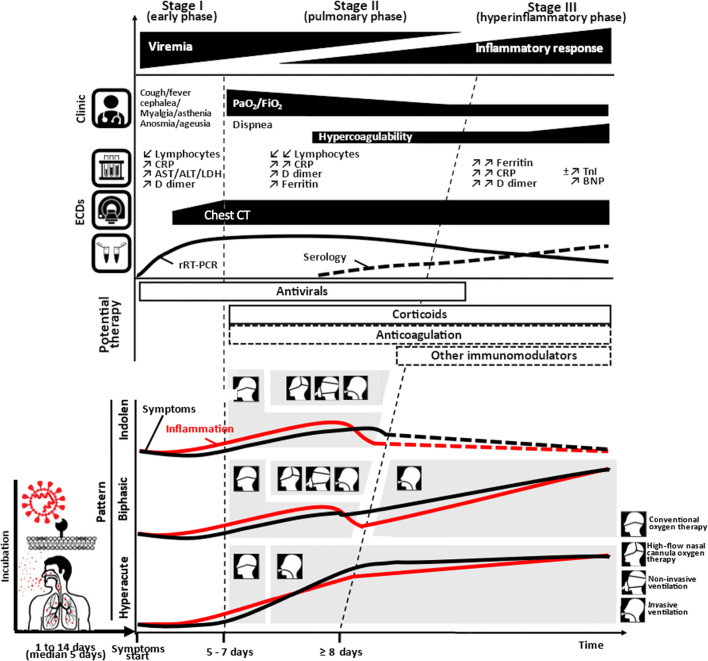
COVID-19 stages and potential specific and supportive therapies.

After an incubation period (time from exposure to the onset of symptoms) of 1 to 14 days (median of 5 days), the symptomatic period begins. COVID-19 evolves through three different stages,^(^31^)^ which are not always present (Figure 2 - Appendix 1).

**Stage I (early phase)**, resulting from viral replication, is characterized by clinical stability with mild symptoms. In Portugal,^(^2^)^ the most frequent symptoms are cough and fever, usually in association with myalgia, headache and asthenia. Atypical symptoms are also described, and gastrointestinal and stomatological changes (anosmia and/or ageusia) are the most frequently reported (possibly in isolation) in retrospective studies. There is no specific analytical signature of COVID-19, but lymphopenia (lymphocyte count < 1.0 × 10^9^/L), associated with a slight increase in C-reactive protein, transaminases and lactate dehydrogenase,^(^32^,^44^)^ is frequent during this stage. The amplitude of the analytical changes, namely, the degree of lymphopenia and elevation of D-dimer, in the early stage of the disease is associated with the probability of clinical progression to the later stages of COVID-19.^(^45^)^ The appearance of changes on plain chest radiography is unlikely, but there are studies showing that changes in chest computed tomography (CT) may precede positivity identified via molecular biology tests.^(^46^)^

**Stage II (pulmonary phase)**, resulting from adaptive immune response activation and thrombo-inflammatory activity, which result in tissue injury with predominantly pulmonary presentation, typically begins 5 to 7 days after the onset of symptoms, coinciding with the time of hospitalization.^(^47^)^ In Portugal,^(^48^,^49^)^ similar to the rest of the world,^(^44^)^ these patients tend to be older (> 60 years) with more cardiovascular risk factors (hypertension, diabetes mellitus and, in particular, obesity) and comorbidities (heart disease, chronic kidney disease and neuromuscular disease). Clinical presentation is more frequently due to worsening of the respiratory condition, with cases of silent hypoxemia being described,^(^50^)^ with an absence of dyspnea (due to normal pulmonary compliance), although with increased respiratory drive,^(^35^,^36^)^ typically with radiological images characterized by focal or multifocal peripheral ground-glass infiltrates with predominantly basal or, later, crazy-paving pattern, and symptomatic hypoxemia being described, with clear signs of increased respiratory effort (due to reduced pulmonary compliance), typically with radiological images characterized by a confluence of consolidations predominantly affecting the dependent areas of the lung. Progression in this stage is characterized by a gradual increase in C-reactive protein, and the first manifestations of coagulopathy associated with COVID-19 may occur, characterized by increases in D-dimer and fibrinogen.^(^30^)^

**Stage III (hyperinflammatory phase)**, resulting from dysregulated immune responses conditioning cytokine storm syndromes, usually occurs during hospitalization and is characterized, from the clinical point of view, by severe worsening of the respiratory condition (invariably with the need for ventilatory support), often associated with hemodynamic instability and multiorgan insufficiency. Although the analytical markers of the hyperinflammatory phase are nonspecific, extreme elevation of C-reactive protein associated with elevation of D-dimer and ferritin is frequent.^(^51^,^52^)^ Although its diagnostic relevance is questionable, proinflammatory ILs, namely, IL-6, are also elevated.^(^53^)^ Once again, it is extremely important to recognize that the diagnosis of the hyperinflammatory phase implies the exclusion of bacterial overinfection (for which procalcitonin may play an important role)^(^33^)^ because there is often a temporal coincidence between the two. In addition, patients do not progress through all three stages, and the rate of onset of respiratory failure is variable.^(^54^)^ Other markers (for example, troponin I and natriuretic peptide type B) may be used in conjunction with other complementary diagnostic tests (for example, echocardiography) for the diagnosis of specific organ involvement.^(^40^)^

The rate of onset of respiratory failure^(^54^)^ may occur with **hyperacute presentation** (fulminant form, progressing in hours), **indolent presentation** (progressive form, progressing in days) or **biphasic presentation** (initially indolent form followed by clinical improvement and subsequent reaggregation). Hyperacute presentation in the first 7 days of disease progression is atypical and requires the exclusion of a previously existing disease (for example, decompensation of congestive heart failure), which has important therapeutic and prognostic implications.

**Long-term COVID-19** includes pulmonary sequelae as well as extrapulmonary sequelae (cardiovascular, neurological and/or psychological), and only limited information is available regarding clinical presentation and long-term prognosis.^(^43^)^

### Extrapulmonary manifestations

#### Hematological manifestations

Patients infected with SARS-CoV-2 may present several hematological changes, and lymphopenia is a cardinal laboratory finding (in up to 90% of patients),^(^32^,^44^,^55^)^ with a reduction in CD4+ and CD8+ lymphocyte subpopulations associated with worse clinical progression.^(^56^)^ Leukocytosis with neutrophilia (rarer) is also a marker of poor prognosis.^(^32^,^44^,^55^)^

Coagulopathy associated with COVID-19, with the previously described analytical pattern, is essentially a prothrombotic dyscrasia (the hemorrhagic risk in COVID-19 is relatively low, approximately 2.7%),^(^57^)^ with strong variability in the incidence and distribution of events, depending on the thromboprophylaxis regimens.^(^57^,^58^)^ COVID-19 is associated with venous thromboembolic events (cumulative incidence of deep vein thrombosis and pulmonary thromboembolism, 31.3%),^(^58^)^ even under prophylactic anticoagulation;^(^57^)^ arterial thrombotic events (for example, acute myocardial infarction and stroke);^(^59^)^ intravenous catheter thrombosis and coagulation of extracorporeal systems (for example, hemofilters in the context of the renal function replacement technique);^(^57^)^ and microthrombotic phenomena, which contribute to the pathophysiology of hypoxemic respiratory failure.^(^38^)^ Thrombocytopenia and increased D-dimer concentrations upon admission (and longitudinal increase during hospitalization) are associated with more severe disease and a worse prognosis.^(^55^,^60^)^

#### Gastrointestinal and hepatobiliary manifestations

The gastrointestinal manifestations of SARS-CoV-2 infection (anorexia, nausea/vomiting, diarrhea and abdominal pain) are frequent (up to 60% of patients) and may occur in isolation. Viral shedding in feces is frequent, and the presence of gastrointestinal manifestations is associated with longer disease duration but not with clinical severity.^(^61^)^ Importantly, gastrointestinal bleeding is rare in the context of COVID-19, even in critically ill patients under mechanical ventilation and with coagulation disorders.^(^62^)^ Hepatocellular lesions (with elevation of transaminases less than five times the upper limit of normal) is frequent in SARS-CoV-2 infection, and there is an association between the magnitude of liver function changes and disease severity.^(^61^)^

#### Cardiovascular manifestations

SARS-CoV-2 infection can result in direct and indirect cardiovascular injury,^(^40^,^63^)^ i.e., ischemic cardiac injury (including type 1 acute myocardial infarction due to atherothrombotic coronary disease and precipitated by atherosclerotic plaque disruption and type 2 acute myocardial infarction due to an imbalance between oxygen supply and needs) and nonischemic (infectious myocarditis) or inflammatory injury potentially associated with ventricular dysfunction (left or global) complicated by acute heart failure or cardiogenic shock;^(^40^,^63^)^ acute cor pulmonale, associated or not with pulmonary thromboembolism;^(^64^)^ and dysrhythmias, including a higher prevalence of de novo atrial fibrillation and prolonged QTc since admission.^(^65^)^ The frequency and magnitude of the elevation of cardiac biomarkers (for example, troponin and natriuretic peptide type B) are associated with more severe disease and a worse prognosis, especially in patients with preexisting cardiovascular disease.^(^66^)^

#### Neurological manifestations

Mild neurological manifestations of SARS-CoV-2 infection (headache, dizziness and myalgia) are frequent (up to 50% of patients),^(^67^)^ and stomatological manifestations (especially ageusia and anosmia) may occur in isolation (in up to 3% of patients).^(^68^)^ Severe neurological manifestations of COVID-19 can occur by different direct and indirect mechanisms and are varied: acute stroke (in up to 6% of critically ill patients); alteration of the state of consciousness; acute inflammatory demyelinating polyneuropathy (Guillain-Barré-like syndrome); posterior reversible encephalopathy syndrome (PRES); and acute necrotizing encephalopathy, including the brainstem and basal ganglia.^(^40^)^

#### Renal manifestations

Acute kidney injury, resulting from glomerular and/or tubular pathology (by direct or indirect mechanisms), is a frequent complication (up to 30% of patients) of SARS-CoV-2 infection,^(^69^,^70^)^ affecting up to 22% of those admitted to intensive care units.^(^65^)^ Changes in urinary sediment (for example, proteinuria and hematuria) are frequent (up to 90% of patients),^(^71^)^ and the elevation of serum biomarkers of acute kidney injury (for example, creatinine) is associated with increased mortality.^(^70^)^ A history of end-stage renal disease (especially patients on hemodialysis and renal transplant recipients) is associated with more severe disease and a worse prognosis.^(^72^)^

#### Endocrine manifestations

Patients hospitalized with COVID-19 often present changes in glucose metabolism, especially euglycemic ketosis and diabetic ketoacidosis in addition to hyperglycemia,^(^40^)^ and changes in thyroid function, namely, reductions in thyroid-stimulating hormone (TSH) and FT4.^(^73^)^ A history of diabetes mellitus and/or obesity is associated with more severe disease and a worse prognosis.^(^74^)^

#### Dermatological manifestations

Mild (acrocutaneous lesions (leg), maculopapular rash (urticaria) and papulovesicular rash) and greater severity (exanthema and livedo reticularis) dermatological manifestations are frequently found in patients hospitalized with COVID-19 (up to 20% of patients), either at admission or during the disease course.^(^75^)^

#### Definitions of severity

The definitions of COVID-19 severity by the WHO^(^76^)^ provide a pragmatic structure (based on clinical indicators) to define subgroups of disease severity. We used these criteria (Table 1 - Appendix 1) as aids in the evaluation, guidance and treatment of patients with COVID-19.

## DIAGNOSIS OF INFECTION

### Diagnosis of SARS-CoV-2 infection

**Table:** 

It **is recommended** that all patients requiring hospitalization in intensive care units undergo a diagnostic test to identify SARS-CoV-2.
It **is recommended** that the initial diagnostic test in patients requiring hospitalization in intensive care units be a molecular nucleic acid amplification test (NAAT) using a sample from the upper respiratory tract (exudate from the nasopharynx and oropharynx collected with a swab) in the context of pneumonia, whenever possible, to the lower respiratory tract (for example, bronchial secretions collected by endotracheal aspirate).
It **is suggested** that when NAAT results cannot be obtained in less than 12 hours (or if NAATs are not available), a rapid antigen test should be used, and a confirmatory NAAT should be conducted as soon as possible if the rapid antigen test result is negative.
It **is suggested** that during hospitalization, between the third and fifth day after the initial negative test and periodically every 5 days (counted from the last test), NAATs should be used for screening.
It **is recommended** *not* to use serological tests in the acute phase.
It **is recommended** *not* to use chest CT as the first diagnostic test in patients with suspected SARS-CoV-2 infection.

The primary aims of laboratory diagnostic tests for SARS-CoV-2 are to diagnose COVID-19 and to detect asymptomatic or presymptomatic cases, limiting the spread in the hospital setting.^(^77^)^ Molecular NAATs (reverse transcription-polymerase chain reaction (RT-PCR) and real-time RT-PCR (rRT-PCR)) are the reference methods (gold standard) for the diagnosis and screening of SARS-CoV-2 infection.^(^77^)^

The NAATs for the identification of SARS-CoV-2 have high specificity, and patients with a higher viral load (further into the disease course) may be more likely to have a positive test. However, in patients with suspected COVID-19 and an negative initial rRT-PCR result, repetition (conversion over days) was positive in 23% of cases (with 4% more cases identified by a third test), indicating a sensitivity < 80%.^(^46^)^ This means that a single negative rRT-PCR test does not exclude SARS-CoV-2 infection.

Rapid antigen tests are proximity tests (point-of-care), with results available 15 to 30 minutes and an analytical sensitivity (≥ 90%) and specificity (≥ 97%) lower than those for NAATs.^(^78^)^ The only reason for their use should be in the context of the unavailability of the gold standard.

Serological tests assess the immune response to SARS-CoV-2 infection. In viral infections, the immune response lags at least 5 to 7 days from the viremia phase,^(^79^,^80^)^ which is why serological tests are considered inadequate for the evaluation of SARS-CoV-2 infection in the acute phase.^(^81^)^

There are radiological changes suggestive of COVID-19, and studies have shown that changes in chest CT precede NAAT positivity.^(^46^)^ However, the widespread use of CT devices potentially increases the risk of cross-infection and should be reserved for situations that would result in changes in clinical management.^(^82^)^

### Diagnosis of co-infection and superinfection

**Table:** 

The collection of blood cultures (at least two sets of aerobic and anaerobic blood cultures) from the lower respiratory tract is **recommended** for the investigation of other microbiological agents and antigenuria for *Legionella pneumophila* and *Streptococcus pneumoniae*.
It **is suggested** to consider requesting other tests (for example, NAATs for other viruses, e.g., influenza, and other respiratory viruses, serology for atypical microorganisms, galactomannan detection) based clinical symptoms and epidemiology.

Coinfection by other microbiological agents, especially in the presence of septic shock, is possible.^(^83^)^ Patients with suspected or confirmed SARS-CoV-2 infection should undergo testing, when appropriate, for other agents (bacteria, viruses or fungi). In the context of sepsis, the collection of blood cultures (at least two sets of aerobic and anaerobic blood cultures) and lower respiratory tract samples are indicated for the investigation of other microbiological agents and the detection of antigenuria for *Legionella pneumophila* and *Streptococcus pneumoniae*.^(^83^)^ In an appropriate clinical-epidemiological context, the request for other microbiological tests is indicated (for example, detection by molecular biology methods for influenza virus and other respiratory viruses and serology for atypical microorganisms).^(^84^)^ Coinfection with *Aspergillus* spp. has also been described, and galactomannan assays can be considered in an appropriate clinical-epidemiological context.^(^85^)^

### Criteria for admission to intensive care units

**Table:** 

**It is recommended** that patients with severe or critical COVID-19 criteria be referred early to intensive care units.
**It is recommended** that admission to the intensive care unit be based on a case-by-case assessment that includes the presentation and severity of acute disease, the reversibility and favorable prognosis of acute disease, history of comorbidities, and poor functional status and frailty prior to the acute situation motivating admission.
**It is recommended** that whenever there is no possibility of a local response, referral and transfer of the patient should be based on the intensive care referral network so that the necessary care can be provided.
**It is recommended** that the decision to admit (or not) be accompanied by the development of a care plan based on a decision model shared with the patient or with his or her family; collegial methodology, ideally multiprofessional and multispecialty, coordinated by an experienced intensivist; and the use of national and international standards and guidelines.

The essential part of the decision-making process for admission to intensive care is based on expectations of individual benefits (vital and functional) and on the clinical evaluation of each patient, in his or her biopsychosocial dimension, determining adequate severity stratification and consequent decisions regarding the level of care, ensuring that there is no difference between the necessary care and care provided.^(^86^)^ Decisions of nonadmission to intensive care units should never be confused with abandonment, requiring, on the contrary, the development of a care plan of which an intensivist is an integral part.^(^87^)^

The lack of planning in situations of potential scarcity of resources leads to inefficiency, waste and the use of prioritization and rationing strategies that are otherwise unnecessary. The use of objective criteria favors a better decision; mitigates the anguish and individual discomfort of professionals;^(^7^)^ and attenuates subjectivity and promotes a decision model that involves the patient, their representatives^(^88^)^ and society while maintaining respect for the principle of autonomy.^(^89^)^ The fundamental values that underlie the ethical decision-making matrix of the flow and admission of patients to intensive care units include (1) **planning**, which involves the development and implementation of a proactive contingency plan (developed by intensive care specialists, agreed upon by other hospital services and approved by a board of directors), with a level of interinstitutional collaboration, namely, referral and regional and interregional transfer based on an intensive care medicine referral network;^(^2^)^ (2) **maximizing benefits**, considering four fundamental criteria, i.e., presentation and severity of acute disease, especially the number and severity of organ dysfunctions (for example, Sequential Organ Failure Assessment (SOFA)); the reversibility and prognosis of acute disease; a history comorbidities; and poor functional state and frailty (clinical frailty scale) prior to the acute situation motivating admission;^(^87^,^90^)^ (3) **exercising collegiality and utilizing a shared decision model**, involving the elaboration of a care plan, based on a decision model shared with the patient or his or her family members that represents the patient’s values;^(^91^)^ collegial methodology, ideally involving multiple professionals and multiple specialties, coordinated by an experienced intensivist;^(^86^,^90^,^92^)^ and the use of national and international standards and guidelines;^(^86^,^90^,^92^)^ (4) **imparting equity**, operationalized to avoid *first come, first served*, which cannot be used in situations where the response must be urgent and fast and the lack of resources can be fatal;^(^86^)^ (5) **providing triage and establishing duty of care**, recognizing that in situations of high demand, screening decisions are essential to define the level of care, initiate organ support therapy, recognize therapeutic ceilings, suspend organ support and/or refer patients to palliative care; and (6) **establishing cross-sectional resource use criteria**, not allowing discrimination (positive or negative) in the criteria for resource allocation or the formulation of ethical decisions for patients with COVID-19 or other clinical conditions.

## INFECTION CONTROL

### Personal protective equipment

**Table:** 

It **is recommended** that all health professionals involved in the provision of clinical care to patients with (or suspected of) infection by coronavirus severe acute respiratory syndrome 2 (SARS-CoV-2) use universal protection, contact protection and droplet protection. These measures include hand hygiene and the use of specific, disposable (single use) and waterproof personal protective equipment: surgical mask, eye protection, cap, smock, clean gloves (covering the cuff) and footwear protection (ideally, waterproof shoes and exclusive use in isolation areas or, optionally, waterproof shoe covers).
It **is recommended** that all health professionals involved in the provision of potentially aerosol-generating clinical care (for example, intubation, secretion aspiration, and bronchoscopy) or prolonged contact (> 15 minutes) and/or intimate contact (for example, placement of a central venous catheter, surgery, and cardiopulmonary resuscitation maneuvers) to patients with (or suspected of) SARS-CoV-2 infection use airway protection. These measures include hand hygiene and the use of specific, disposable (single use) and waterproof personal protective equipment: respirator with a facial filter, eye protection (with side protection), cap, smock (with cuffs that tighten or with elastics and that cover up to the middle of the leg or ankle) and apron, clean gloves (covering the cuff of the gown) and footwear protection (ideally waterproof shoes and exclusive use in isolation areas or, optionally, waterproof shoe covers).
It **is suggested** that full protection (waterproof, with built-in hood and neck protection) be limited to professionals with training and practical experience for this purpose.
It **is suggested** that an order and technique for placement (donning) and removal (doffing) of personal protective equipment be strictly adhered to (ideally using a mirror or surveillance by another health professional), ensuring proper sealing of the face mask, with additional care during the removal procedure to avoid contamination of oneself, others and the environment.
It **is recommended** that all health professionals involved in the provision of clinical care have training and practical experience in the procedures for donning and doffing personal protective equipment prior to contact with patients.

Regarding personal protective equipment, the recommendations of the DGS are adopted,^(^93^)^ which are based on the guidelines issued by the WHO^(^94^)^ and the European Center for Disease Prevention and Control (ECDC)^(^95^)^ for the prevention and control of infections in cases of suspected or confirmed infection by SARS-CoV-2.

It is necessary to provide detailed definitions for some medical devices. Surgical masks are used to protect health professionals from exposure to agents transmitted by droplets (large respiratory particles > 0.5µm); respirators with facial filters (which include the N95 or FFP2 and FFP3 masks, depending on the American or European designation and respective filtration rate) are used to protect health professionals from exposure to transmissible agents by air (small respiratory particles, < 0.5µm) or by droplets.

The current evidence, which includes randomized and controlled studies, systematic reviews and meta-analyses of seasonal respiratory viral infections (for example, influenza) and seasonal coronavirus,^(^96^-^98^)^ notes the absence of additional benefits from the use of respirators with facial filters (in relation to masks) by health professionals involved in the provision of nonaerosol-generating clinical care. Regardless of the type of equipment, there is evidence that ensuring a good fit of the mask to the face is an effective way to optimize effectiveness.^(^99^)^

### Organization of services

**Table:** 

**It is recommended** that the management of all level 2 (intermediate) and 3 (intensive) patients in the hospital (regardless of the service in which they are located) be performed by intensive care unit specialists in strict coordination with the Clinical Management, Directorate-General of Health and Ministry of Health.
**It is recommended** that in hospitals where there is more than one intensive care unit, a cohort area of confirmed critical cases of COVID-19 be created and a cohort area of suspected critically ill patients (for transient hospitalization) be considered, namely, establishing criteria for activation.
Isolation in a single room with negative pressure, a shower, private bathroom and adequate ventilation system, with capacity for at least 6-12 air changes/hour, **is recommended**. Once these resources are exhausted, it **is recommended** that patients be isolated in a single room with a ventilation system capable of at least 6-12 air changes/hour. When individual isolation rooms are not available, isolation in a cohort **is recommended**, respecting a minimum distance greater than 1 m between patients.
The delimitation of risk areas and predefined routes for professionals, patients and waste **is recommended**.
**It is recommended to** restrict visitations to all patients and limit the number of professionals in contact with patients (ideally with dedicated professionals), with the implementation of alternative, remote ways of communication between patients and families and between clinical teams, patients and families, regardless of the place of isolation.

Intensive care beds represent a scarce good, and very few exist in individual rooms with negative pressure.^(^100^)^ In this context, it is especially necessary to have a full understanding of the organization and structure of intensive care units to ensure responsiveness and minimize the risk of nosocomial transmission of SARS-CoV-2.

After utilizing all individual rooms with negative pressure, the following strategy should be applied: create COVID-19 and non-COVID-19 cohorts, and within the COVID-19 cohorts, create two subcohorts, i.e., critical suspected COVID-19 cases and critical confirmed COVID-19 cases.^(^101^)^ The benefits resulting from the concentration of experience and resources provided to COVID-19 cohorts should be tempered by detailed attention to the logistical and organizational/structural aspects of these spaces, with simulation training playing an important role in the preparation of the teams, ensuring proficiency in different procedures related to intensive medicine and infection control.^(^102^)^

From the outset, clear delimitation of risk areas is necessary.^(^103^)^ **Areas of basal risk (clean or green areas)** are areas where circulation occurs in accordance with the rules of the rest of the hospital and where all support services should be moved (for example, information systems for clinical practice, pharmacy and storage spaces). **Intermediate-risk areas (gray areas)** are transition zones between basal risk areas and high-risk areas, and **high-risk areas (red areas)** are areas where direct care is provided to patients, requiring the use of appropriate personal protective equipment and where the possibility of placing the entire space under pressure should be evaluated (maximizing the number of air recirculations).

The transition between these different areas should be made through predefined routes for professionals, patients and waste. In the specific areas accessed by professionals, zones (ideally equipped with mirrors) should be created for the donning and doffing of personal protective equipment, with proximity to bathing areas. Waste corridors must be separate from all other walkways, in compliance with the appropriate guidelines (accommodation, cleaning and transportation of properly identified containers).

The implementation of alternative, remote ways of communication between patients and families and between treatment teams, patients and families has benefits for patients and families and increases the quality of care.^(^104^)^

## SUPPORTIVE THERAPY

### Oxygen therapy, respiratory support and adjuvant therapies

**Figure 3:**
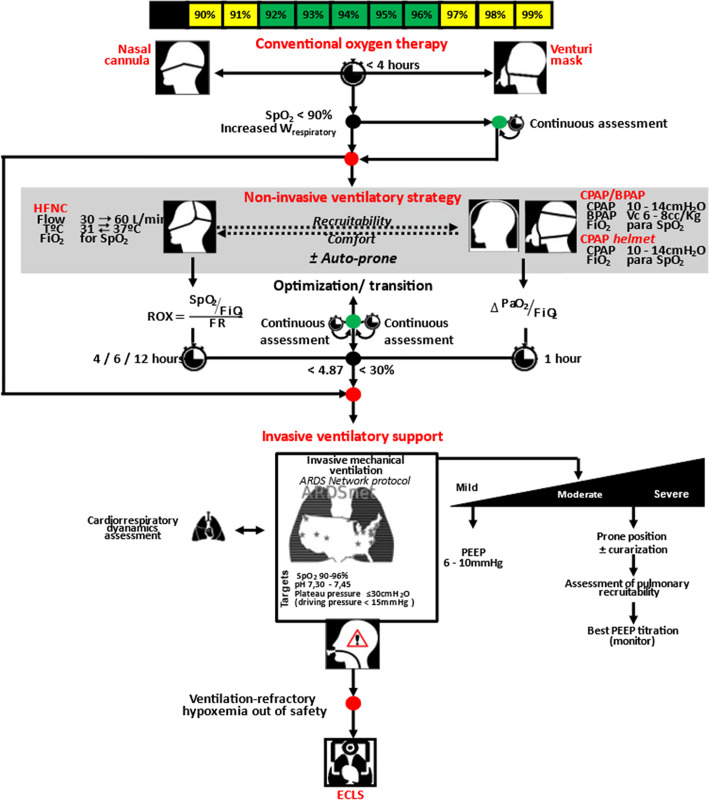
Strategy of oxygen therapy and ventilatory support in respiratory failure due to COVID-19.

The indications for oxygen therapy and respiratory support for respiratory failure in the context of COVID-19 are summarized in figure 3 (Appendix 1).

**Table:** 

In patients with COVID-19, it **is recommended** to administer conventional oxygen therapy (through a nasal cannula or a Venturi mask) if peripheral oxygen saturation (SpO_2_) < 90%, with the goal of an SpO_2_ between 92% and 96%.
When using nasal cannulae, it **is suggested** to place a surgical mask over the oxygen supply device. When using a Venturi mask, a device that incorporates a filtering medium in the exhalation ports or, optionally, the placement of a surgical mask under the oxygen supply device **is suggested**.

**Conventional oxygen therapy** should be administered if peripheral oxygen saturation (SpO_2_) is less than 90%, with the goal of an SpO_2_ between 92% and 96%, through a nasal cannula or Venturi mask.^(^76^)^ There are no specific data on COVID-19, but in critically ill patients, hypoxemia,^(^105^)^ as a liberal oxygen therapy strategy,^(^106^)^ is associated with worse outcomes (including mortality); therefore, an SpO_2_ between 92% and 96% is considered reasonable.^(^76^,^98^)^ To reduce the risk of aerosolization, a surgical mask can be placed over the nasal cannula, or, in the case of using a Venturi mask, choose a device that incorporates a filtering medium in the exhalation ports (for example, Intersurgical FiltaMask™),^(^107^)^ or optionally, place a surgical mask under the oxygen supply device.^(^108^)^ No humidification is required for oxygen flows < 4L/minute,^(^109^)^ and the use of bubble humidifiers with oxygen flow ≥ 5L/minute potentially produces aerosols with a risk of microorganism transmission.^(^110^)^

**Table:** 

It **is suggested**, in patients with COVID-19, in the failure of conventional oxygen therapy (peripheral oxygen saturation-SpO_2_ < 92% with fraction of inspired oxygen (FiO_2_) > 0.6, increased respiratory work and/or respiratory rate ≥ 30 cpm) consider, in the absence of criteria for endotracheal intubation, a trial of non-invasive ventilatory therapies (high-flow nasal cannulae (HFNC) or noninvasive mechanical ventilation (NIV)) provided that (1) professionals use contact, droplet and airway precautions (ideally in rooms or areas with negative pressure) and strategies aimed at minimizing aerosol production are used; (2) a protocol suitable for respiratory failure is established and implemented; (3) the technique is initiated in a highly monitored environment to avoid delays in endotracheal intubation in the event of failure of response; and (4) failure criteria are established and respected.

When failure of conventional oxygen therapy (SpO_2_ < 92% with FiO_2_ > 0.6, increased respiratory work and/or respiratory rate ≥ 30 cpm) should be considered, in the absence of criteria for endotracheal intubation, noninvasive ventilatory therapies (HFNC or NIV) should be attempted.

The use of HFNC and NIV in respiratory failure due to COVID-19 was initially disputed due to a concern associated with the potential creation and propulsion of droplets and/or aerosols, with a risk of in-hospital transmission, particularly to health professionals.^(^111^)^ Regarding HFNC, the best evidence demonstrates that the risk of aerosol generation is low (not higher than conventional oxygen therapy) when HFNC is correctly applied (with adapted nasal cannulae).^(^112^,^113^)^ NIV was systematically associated with an increased risk of aerosol generation,^(^22^,^111^)^ especially when using ventilated and/or poorly sealed oronasal masks combined with single-circuit ventilators. These techniques should ideally be performed in negative pressure rooms (with at least six air changes per hour) or in rooms equipped with HEPA (high efficiency particulate air) filters or, in the absence of these conditions, in rooms with natural ventilation with an air flow of at least 160 L/second per patient.^(^114^)^ HFNC and NIV should not be excluded based only on the risk of in-hospital transmission, especially if professionals use contact, droplet and airway precautions. In addition to the classic criteria for invasive mechanical ventilation (respiratory or cardiocirculatory arrest, hemodynamic instability and altered state of consciousness), the choice of a ventilatory therapy trial is justified as long as a protocol suitable for respiratory failure is established and implemented; the technique is initiated in an environment of high monitoring, avoiding delays in endotracheal intubation in case of failure of response; and failure criteria are established and respected. Respecting these criteria, in hypoxemic acute respiratory failure in the context of COVID-19, a late invasive mechanical ventilation strategy (compared to an early strategy) has not been associated with increased mortality or other relevant outcomes,^(^115^-^117^)^ and in some series, it was also associated with a reduction in mortality.^(^118^)^

**Table:** 

It **is suggested** that the choice between noninvasive ventilatory therapies (HFNC and NIV)) is based on weigh individual risks and benefits as well as on the availability of equipment/interfaces and local experience of the staff.

Aside from carbon dioxide retention (partial pressure of carbon dioxide (PaCO_2_) > 45mmHg in the context of acute respiratory failure), there is currently no evidence of superiority between HFNC and NIV for respiratory failure due to COVID-19. Thus, until defining clear phenotypes of respiratory failure associated with COVID-19, the decision should be based on weighing individual risks and benefits (for example, the use of HFNC may allow for more comfort, while the use of NIV may be more beneficial in patients with an obese biotype and/or evidence of alveolar collapse on chest imaging because it allows alveolar recruitment)^(^107^)^ and on the availability of equipment/interfaces and experience of the staff.

**Table:** 

It **is suggested** that if a decision to use high-flow oxygen therapy via nasal cannula is made (1) a surgical mask should be placed over the nasal cannulae; (2) nasal cannulas should be adapted to the size of the nostrils, with a flow rate of 50 - 60L/minute and FiO_2_ titrated for SpO_2_ between 92% and 96%; (3) the ROX index should be evaluated at 2, 6 and 12 hours, with maintenance of support if ≥ 4.88, in the absence of criteria for endotracheal intubation; and (4) in case of failure, treatment should be optimized, considering increased support up to 60L/minute in a prone position, a transition to NIV or endotracheal intubation (and invasive ventilatory support).

The application of **HFNC** therapy uses nasal cannulas (which should occupy ≥ 50% of the size of the nostrils), starting with flows of 20 - 30L/minute, which can be increased (at levels of 10L/minute in short intervals) up to 50 - 60L/minute,^(^107^)^ to provide an average positive end-expiratory pressure (PEEP) of 5 - 6cmH_2_O (with mouth closed). The temperature (initially 37°C) is titrated based on the patient’s preferences and secretion characteristics, and FiO_2_ is titrated for SpO_2_ between 92% and 96%. HFNC therapy can be performed using dedicated systems with turbines (connected to an oxygen source) and conventional fans with active humidification systems, in addition to flow meters (high flow rate, connected to an air and oxygen source) and mixers combined with the active humidification system.

The risk of aerosolization is reduced when a surgical mask is placed over the nasal cannula.^(^113^)^

HFNC therapy, in the context of hypoxemic acute respiratory failure, has shown improved results,^(^119^-^121^)^ and there is evidence of its efficacy in patients with COVID-19,^(^122^)^ with a higher success rate when the initial PaO2/FiO2 ratio is > 200mmHg.^(^123^)^ This evidence led the panel of experts of the Surviving Sepsis Campaign to recommend HFNC as the primary noninvasive ventilatory therapy.^(^98^)^

There are validated scores for hypoxemic respiratory failure (ROX index and SpO_2_/FiO_2_/respiratory rate)^(^124^)^ that have been validated for COVID-19.^(^125^-^127^)^ Although the failure criteria are slightly different for COVID-19, the ROX index should be evaluated at 2, 6 and 12 hours, maintaining the therapy if ≥ 4.88 in the absence of criteria for endotracheal intubation. Lower values should be considered potential failure and should lead to therapy optimization, considering increased support up to 60 L/minute in the prone position, a transition to NIV or endotracheal intubation (and invasive ventilatory support).

**Table:** 

It **is suggested** that if noninvasive mechanical ventilation is initiated, (1) interfaces with maximum sealing should be used, as well as specific ventilators and ventilatory circuits with antibacterial/antiviral filters; (2) ideally, non-invasive ventilation (NIV) helmets or, optionally, face masks (or oronasal) capable of specific configurations for continuous positive airway pressure (CPAP; up to a maximum of 12 - 14cmH_2_O) or bilevel positive airway pressure (BPAP; with support pressure to maintain tidal volume between 6 and 8mL/kg), FiO_2_ titrated to SpO_2_ between 92% and 96% should be used; (3) PaO_2_/FiO_2_ should be evaluated at 1 hour with maintenance of support and improvement (ΔPaO_2_/FiO_2_) ≥ 30%, in the absence of criteria for endotracheal intubation; and (4) in case of failure, therapy should be optimized, considering increased support in a prone position, eventual transition to HFNC therapy in a prone position or endotracheal intubation (and invasive ventilatory support).

The application of **NIV** involves the administration of CPAP/PEEP with initial continuous pressures of 8 - 10cmH_2_O to a maximum of 12 - 14cmH_2_O (with the need to compensate the resistance imposed via the use of a heat and moisture exchange filter (HMEF) and antibacterial/antiviral filters), using multiple interfaces and ventilation systems that provide FiO_2_ of 0.8 - 1.0.^(^107^)^ The association of positive pressure during the inspiratory phase (IPAP) greater than that applied during the expiratory phase (EPAP) provides complete ventilatory support, i.e., bilevel positive airway pressure *(*BPAP). The pressure support corresponds to the difference between the IPAP and EPAP (often called DP) and should be adjusted to maintain a tidal volume between 6 and 8mL/kg.^(^107^)^

The risk of aerosolization can be minimized using helmets or interfaces with maximum sealing, as well as ventilators and ventilatory circuits with antibacterial/antiviral filters.^(^128^)^

NIV can be applied via different interfaces: ideally, VNI helmets with air cushions, or, optionally, face masks (or oronasal masks) not ventilated (without intentional leakage and without an anti-asphyxia valve). When applying the helmet-type interface, the specific configurations used should be different from those for facial (or oronasal) masks, with an increase in pressures (IPAP/EPAP) by 50% as well as an increase in the pressurization rate.^(^129^)^ These interfaces connect to dual circuit systems in dedicated ventilators or conventional intensive care ventilators, to single circuits with passive exhalation valves incorporated in the circuit or added to the circuit (for example, whisper swivel or plateau valves), or to active exhalation valves (connected to the pressure line and the flow line). The environment should be protected by HMEFs and antibacterial/antiviral filters in double HMEF circuits, placed between the interface and Y, by an antibacterial/antiviral filter, placed in the connection between the expiratory branch and the ventilator, and by single HMEF circuits, placed between the interface and the ventilator interface and the exhalation valve and antibacterial/antiviral filter, placed between the circuit and the ventilator. Another option is CPAP/PEEP administration with high FiO2 and flow meters (high flow rate, connected to the oxygen source), connected by means of a flow acceleration valve to an interface (oronasal mask; for example, CPAP Boussignac) or a helmet with adjustable PEEP valve in the expiratory branch. Similar to what occurs with NIV circuits, the environment must also be protected by the interposition of HMEFs and antibacterial/antiviral filters placed between the flow acceleration valve and the oronasal mask or in the expiratory branch of the helmet before the PEEP valve.

NIV is indicated in the acute exacerbation of chronic hypercapnic respiratory failure (for example, pulmonary obstruction, obstructive sleep apnea and obesity-hypoventilation syndrome),^(^130^)^ in which the use of BPAP is promoted, and hypoxemic acute respiratory failure associated with acute cardiogenic lung edema,^(^131^)^ in which the use of CPAP/PEEP is promoted. In hypoxemic acute respiratory failure, the evidence suggests that the use of NIV with a helmet (but not with a face mask) produces better results (reduction in the risk of endotracheal intubation and mortality benefits) than HFNC,^(^121^)^ and for COVID-19, there are data that NIV using a helmet, compared to HFNC, reduces the risk of intubation.^(^132^)^ These data come mainly from Italian groups who have substantial experience in the use of NIV with a helmet (which has a long learning curve) and cannot be generalized to low-volume centers.

Although there is a validated score to evaluate hypoxemic respiratory failure in NIV, the HACOR index,^(^133^)^ it has not yet been validated in the specific context of COVID-19. Maintenance of support is suggested if PaO_2_/FiO_2_ improves ≥ 30% afer 1 hour compared to PaO_2_/FiO_2_ prior to the onset of NIV (which indicates pulmonary recruitability),^(^134^)^ in the absence of criteria for endotracheal intubation. Lower values should be considered potential failure and should lead to therapy optimization, considering increased support in a prone position, eventual transition to NIV in a prone position or endotracheal intubation (and invasive ventilatory support). Additionally, an expired tidal volume > 8 mL/kg of ideal weight is a predictor of NIV failure^(^135^)^ and should lead to the consideration of endotracheal intubation (and invasive ventilatory support) because it is associated with changes in the pressure gradient, potentially resulting in P-SILI.^(^34^)^ In patients with NIV, rotation of noninvasive ventilatory strategies should be considered, with periods of up to 1 hour of HFNC therapy, allowing oral feeding and rest.^(^107^)^

**Table:** 

A structured prone protocol (when awake) **is suggested** for all patients under HFNC therapy or NIV able to comply with orders, as long as clinically tolerated.

A **prone** position (ventral decubitus, when awake) may, in combination with NIV or HFNC therapy, increase comfort and improve PaO_2_/FiO_2_ by up to 35mmHg,^(^136^)^ a benefit already demonstrated in patients with COVID-19.^(^137^,^138^)^ An increasing number of observational studies (not randomized controlled studies) describe the safety and clinical benefits (reduction in dyspnea and respiratory work, but with no benefit in mortality or reduction in intubation rate) of prolonged periods (> 16 hours per day) of HFNC therapy in a prone position for hypoxemic respiratory failure due to COVID-19, especially with PaO_2_/FiO_2_ < 300.^(^139^)^ There is no clear protocol for the use of the prone position; however, at least twice a day for periods longer than 30 minutes, until the patient shows fatigue/intolerance, is recommended.^(^136^)^ The protocol (including the provision of an information leaflet), with the establishment of a long-term strategy (for example, variation in position between ventral decubitus, right lateral decubitus, left lateral decubitus and Fowler decubitus, every 2 hours) associated with positioning adjuvants, may be useful for improving therapeutic adherence.^(^114^)^

**Table:** 

A structured protocol for weaning from noninvasive ventilatory therapy **is suggested**.

If clinical and blood gas stability is maintained in NIV and/or HFNC therapy, the **weaning process from NIV therapy** should be initiated. For HFNC therapy, the flow rate should be initially maintained, with a progressive reduction in FiO_2_ of 0.40 until reaching the target SpO_2_.^(^140^)^ A subsequent weaning protocol has not yet been established, but reducing the output by 10 L/minute to 20L/minute (with maintenance of FiO_2_) is recommended, after which the reduction in FiO_2_ can begins until complete autonomization.^(^107^)^ For NIV with a face mask, there is no formal approach to ventilatory weaning. Typically, when withdrawing the interface (for example, for oral feeding), the patient’s tolerance is evaluated. If there are no signs of respiratory distress or worsening of SpO_2_ during this period, NIV can be discontinued. In patients in whom the primary cause has been resolved but who do not tolerate the suspension of NIV, weaning should occur over the course of periods; that is, the clinician should reduce the time to progressively decrease NIV, preferentially maintaining ventilation during sleep. For NIV with a helmet, a spontaneous breathing test (ERT) should be performed if the patient does not show signs of respiratory distress and maintains target a SpO_2_ with FiO_2_ < 0.5 and PEEP ≤ 6cmH_2_O. At least 24 hours with FiO_2_ ≤ 0.4 (by Venturi mask or HFNC) and PaO_2_/FiO_2_ > 250 is considered successful weaning.^(^107^,^134^)^

**Table:** 

It **is suggested** that the decision of endotracheal intubation be based on a composite evaluation of the oxygenation state (as assessed by the ROX index and/or PaO_2_/FiO_2_) and ventilation (respiratory acidosis with pH < 7.30) but also on the respiratory effort perceived by the patient.
We **suggest** a structured protocol for endotracheal intubation, performed by an experienced operator, using contact, droplet and airway precautions (ideally in a negative pressure room).

The need for endotracheal intubation and invasive ventilatory support should be based on the clinical *gestalt* of an experienced intensivist,^(^141^)^ encompassing not only the state of oxygenation (as assessed by the ROX index and/or PaO_2_/FiO_2_) and ventilation (respiratory acidosis with pH < 7.30) but also an evaluation of respiratory effort perceived by the patient (dyspnea or intolerable discomfort) and other factors (for example, unmanageable volume of bronchial secretions). The gold standard for the evaluation of increased respiratory effort is the assessment of the electrical activity of the diaphragm using surface electrodes or esophageal catheters, followed by a quantitative evaluation of inspiratory effort (esophageal pressure), which is neither widely available nor compatible with use at the bedside outside a study environment.^(^142^)^ The evaluation of respiratory effort should be based on an objective examination, e.g., the presence of a rapid breathing pattern (respiratory rate ≥ 30 cpm), thoracoabdominal breathing, accessory respiratory muscle use, including palpation of the sternocleidomastoids, and thoracoabdominal breathing,^(^142^)^ and, eventually, ultrasound evaluation of the diaphragm, namely, evaluation of the diaphragmatic thickening fraction.^(^143^)^

When an endotracheal intubation decision is made (procedure with a high risk of aerosol generation),^(^22^,^111^)^ all strategies that minimize the risk of transmission to health professionals should be used.^(^144^)^ The procedure should be performed by an experienced operator (the operator with the highest probability of intubation on the first attempt) with contact, droplet and airway precautions (ideally in a negative pressure room) and using a systematized protocol,^(^144^)^ as systematized in table 2 (Appendix 1).

**Table:** 

It **is suggested** that after intubation and invasive ventilatory support, the following be used: (1) a classic ventilation strategy based on the ARDS Network protocol (tidal volume of 4 - 6mL/kg of ideal body weight with an upper limit plateau pressure < 30cmH_2_O) with minimum respiratory rate for pH > 7.30 associated with a driving pressure < 15cmH_2_O; (2) ventral decubitus for minimum periods of 16 hours if PaO_2_/FiO_2_ < 150mmHg; (3) neuromuscular blockers for ≤ 48 hours if PaO_2_/FiO_2_ < 150mmHg or severe dyssynchrony or elevated respiratory drive not controlled by optimized analgesics; and (4) in mild ARDS (PaO_2_/FiO_2_ between 200 - 300mmHg), the use of low PEEP, and in moderate to severe ARDS (PaO_2_/FiO_2_ < 200mmHg), application of high PEEP only after an evaluation of recruitment potential.

Once **invasive ventilatory support** is initiated, a protective ventilation strategy associated with adjuvant therapies, both personalized, should be used, guided by clinical, imaging and ventilatory mechanics parameters. The recommendations made are based on international recommendations^(^145^)^ for typical ARDS (not associated with COVID-19), which systematically reduces morbidity and mortality in this population: a classic ventilation strategy based on the ARDS Network protocol (tidal volume of 4 - 6mL/kg of ideal body weight with a limit higher for plateau pressures < 30cmH_2_O) with a minimum respiratory rate at pH > 7.30^(^146^,^147^)^ associated with a driving pressure < 15cmH_2_O;^(^148^)^ and decubitus for minimum periods of 16 hours if PaO_2_/FiO_2_ < 150mmHg^(^149^)^ and neuromuscular blockers for ≤ 48 hours if PaO_2_/FiO_2_ < 150mmHg,^(^150^)^ or severe dyssynchrony or elevated respiratory drive not controlled by optimized analgosedation.^(^35^)^ Respiratory drive can be monitored using P 0.1 (airway occlusion pressure, i.e., pressure generated in the airways during the first 100 milliseconds of an inspiratory effort against an occluded airway), considering a cutoff value > 3.5cmH_2_O for increased respiratory drive.^(^142^,^151^)^ Regarding the PEEP strategy, for mild ARDS (PaO_2_/FiO_2_ between 200 - 300mmHg), the use of a low PEEP without recruitment maneuvers should be considered because there is no clear evidence of benefits (and potential risks) of using a high PEEP strategy (associated or not with recruitment maneuvers),^(^152^-^155^)^ which does not exclude the use of low-risk recruitment maneuvers (for example, CPAP 40/40) after derecruitment maneuvers (aspiration and disconnection). Furthermore, in moderate to severe ARDS (PaO_2_/FiO_2_ < 200mmHg), the ideal PEEP level depends on the ARDS phenotype, which can be assessed by means of recruitment potential based on chest imaging^(^156^)^ or on pulmonary mechanics, reserving high PEEP for cases of potential pulmonary recruitment. Recruitment potential can be measured via ventilatory mechanics (for example, recruitment/inflation ratio)^(^157^)^ followed by the best PEEP trial strategy, based on an approach validated using available resources; if several techniques are available, the best adapted to the characteristics of the patient should be used, e.g., high PEEP based on the ARDS Network protocol; an approach to improve static compliance or driving pressure; a maximum recruitment maneuver followed by PEEP for optimal SpO_2_ or better static compliance; incremental PEEP to achieve a plateau pressure below 30cmH_2_O; or transpulmonary pressure calculated by esophageal manometry.^(^145^)^

**Table:** 

It **is recommended** that routine use of inhaled nitric oxide is not used.

The **use of inhaled nitric oxide** in typical ARDS results in a transient improvement in oxygenation but has no significant effect on mortality and is associated with an increased risk of acute kidney injury.^(^158^)^ Thus, inhaled nitric oxide therapy should not be used routinely; however, the recommendation does not rule out its use as a rescue therapy (i.e., ARDS is associated with refractory hypoxemia), especially if associated with right ventricular dysfunction.^(^159^)^

**Table:** 

A structured protocol for weaning and extubation of invasive ventilatory support **is suggested**.

Regarding **ventilatory weaning**, the need for reintubation associated with progression to the hyperinflammatory stage with a high risk of postextubation respiratory failure have been described (PaO_2_/FiO_2_ > 150mmHg with ≤ 6cmH_2_O with cardiocirculatory stability and an adequate state of consciousness).^(^160^)^ The ERT must be performed at support pressure using a closed circuit (for example, support pressure of 7cmH_2_O for 30 to 120 minutes) and not in a T-tube, which not only minimizes the risk of aerosolization but is also associated with a higher extubation success rate and reduced hospital mortality.^(^161^)^ If tolerance is demonstrated, as assessed by objective and subjective criteria, extubation should be considered.^(^162^)^

The **extubation** procedure, because it is often associated with coughing, is a potentially aerosol-generating procedure, and all strategies that minimize the risk of transmission to health professionals (if extubation occurs during the infectious period of the disease) should be maintained. The procedure should be performed ideally by two operators, with contact, droplet and airway precautions (ideally in a negative pressure room) and using a systematized protocol, as shown in table 3 (Appendix 1).

If **ERT failure occurs**, the patient should be connected to a ventilatory mode that provides comfort and adequate gas exchange, identifying and optimizing potential causes of failure.^(^162^)^ In **difficult weaning** occurs (failure of multiple spontaneous breathing tests), two weaning strategies, successfully studied in randomized clinical trials, are possible: increased ERT time or progressive reduction in support pressure.^(^162^)^

**Table:** 

It **is suggested** to consider tracheotomy from the 10th day of mechanical ventilation.

**Tracheotomy**, similar to the approach for respiratory failure not associated with COVID-19, should be considered from the 10th day of mechanical ventilation.^(^163^)^ The procedure can generate aerosols; therefore, all strategies that minimize the risk of transmission to health professionals (if the procedure occurs during the infectious period) should be maintained. The procedure (percutaneous or surgical) should be performed, ideally, by two operators using contact, droplet and airway precautions (ideally in a negative pressure room) and using a systematized protocol.^(^164^)^

### Bronchofibroscopy and inhalation therapy

**Table:** 

It **is suggested** to reserve bronchofibroscopy for urgent situations (for example, atelectasis with ventilatory impairment and critical obstruction of the central airway) or when the examination results may lead to a significant modification in the therapeutic strategy (for example, suspicion of coinfection or superinfection). It **is suggested** that if a decision is made to perform bronchofibroscopy, the technique should be performed by the most experienced operator, and airway precautions should be used (with, ideally, the procedure occurring in a negative pressure room). Disposable video bronchoscopes and the operator to the rear of the patient’s head **are suggested**.

Bronchofibroscopy is associated with a risk of aerosol generation.^(^22^)^ The indications for use should be selective and always well analyzed, using all strategies that minimize the risk of transmission to health professionals.^(^83^)^ The recommendations contained in the “Position document of the Portuguese Society of Pulmonology for the performance of bronchoscopies during the COVID-19 outbreak” should be followed.^(^165^)^

**Table:** 

It **is suggested** that when the administration of inhalation therapy is clinically indicated, pneumatic, ultrasonic or oscillatory membrane nebulization systems should not be used.

The administration of inhalation therapy using pneumatic, ultrasonic or oscillatory membrane nebulization systems is associated with a risk of aerosol generation,^(^22^)^ and all strategies that minimize the risk of transmission to health professionals should be used.^(^83^,^166^)^

### Extracorporeal life support

**Table:** 

It **is recommended** that critically ill patients with respiratory failure associated with COVID-19 be referred for extracorporeal respiratory support after optimized invasive mechanical ventilation and associated adjuvant strategies fail. It **is recommended** that critically ill patients with cardiogenic shock associated with COVID-19 be referred for extracorporeal cardiorespiratory support when conventional therapy fails.
It **is recommended** that the referral of critically ill patients with respiratory failure and/or cardiogenic shock associated with COVID-19 and indications for extracorporeal life support be restricted to reference centers recognized by the Ministry of Health and the General Directorate of Health.
It **is recommended** that the interhospital transfer of critically ill patients with respiratory failure and/or cardiogenic shock associated with COVID-19 and indications for extracorporeal life support occur within the reference center and be conducted, whenever possible, by an *in loco* dedicated rescue team.

Extracorporeal life support (ECLS), also known as extracorporeal membrane oxygenation (ECMO), is a form of extracorporeal support in which blood is drained by an external pump to a gas exchange membrane (fed by a constant flow of controlled gas via a flow meter equipped with a mixer) and then returned to systemic circulation. There are different forms of ECLS, depending on the blood flow and the cannulation site. High-flow systems, which are of interest in this context, use large-caliber cannulae (18 - 31 F) to drain blood at high flow rates (3.0 to 8.0L/minute) from the venous system and return it to the venous system (veno-venous ECLS, or VV ECLS, which provides respiratory support) or to a large artery (veno-arterial ECLS, or VA ECLS, which provides cardiorespiratory support). Other configurations are also possible, such as veno-arteriovenous (V-AV) ECLS and left ventricular decompression measures (for example, microaxial pumps), which have specific indications.^(^167^)^

The first reports on the use of ECLS in the treatment of patients with severe COVID-19 from China associated the technique with a mortality rate higher than 70%,^(^168^)^ questioning its usefulness, in particular in a pandemic context, in which the optimization of available resources is particularly relevant.^(^169^)^ Additionally, a hypothesis was proposed, according to which ECLS could worsen the prognosis of patients with severe COVID-19 by worsening lymphopenia and exacerbating the inflammatory response resulting from the use of an extracorporeal circuit.^(^170^)^ The European Chapter of the Extracorporeal Life Support Organization (Euro-ELSO) conducted a summary report with weekly updates of COVID-19 cases involving ECLS in Europe that did not confirm these concerns.^(^171^)^

For respiratory failure associated with COVID-19, the type of support to be instituted should be, with particular exceptions, VV ECLS. This modality allows extracorporeal hematosis (oxygenation and removal of carbon dioxide) and has been used in cases of severe respiratory failure refractory to conventional treatment.^(^172^)^ The different configurations (femoro-jugular, jugulo-femoral and femoro-femoral) should be used based on the experience of the reference center and based on the specificities of each patient (for example, presence of deep vein thrombosis and morbid obesity). The use of a single cannula for VV ECLS (for example, cannula), which is not contraindicated, is also not recommended due to the frequent need for high flow in the extracorporeal circuit.^(^167^)^

The indications, as well as contraindications, for referral of critically ill patients with respiratory failure associated with COVID-19 for extracorporeal respiratory support are summarized in table 4 (Appendix 1). In the therapeutic approach, ECLS should only be considered after the failure of optimized invasive mechanical ventilation and associated adjuvant strategies, such as ventral decubitus, neuromuscular block and individualization of ventilatory parameters guided by transpulmonary pressure. The clinical suspicions of pulmonary thromboembolism or patent foramen ovale with a right-to-left shunt should be investigated by means of appropriate imaging tests prior to referral to ECLS.^(^173^,^174^)^

Right ventricular assistance associated with VV ECLS through cannulation of the pulmonary trunk can be considered in the presence of right ventricular dysfunction (after exclusion of pulmonary thromboembolism), and conversion to V-AV ECLS should be considered in the presence of shock associated with severe acute *cor pulmonale*.^(^167^)^

In cardiogenic shock associated with COVID-19, the support modality instituted should be VA ECLS, which allows complete cardiorespiratory support and has been used, based on observational cohorts, in the context of cardiogenic shock (of different etiologies) refractory to conventional treatment.^(^167^)^ A clinical aspect that has received increasing attention and may be relevant in the use of VA ECLS in severe COVID-19 is the description of different forms of cardiac involvement in this disease due to right ventricular dysfunction^(^175^)^ consequent to pulmonary hypertension associated with ARDS^(^175^)^ and acute myocarditis caused by acute SARS-CoV-2 infection.^(^176^)^ In these cases, the emergent use of VA ECLS may constitute a therapeutic option as a bridge to recovery in cases of hemodynamic collapse.^(^177^)^ Due to the frequent incidence of lower limb ischemia associated with arterial cannulation, the use of a return cannula with a lumen ≤ 17 F associated with anterograde reperfusion of the homolateral superficial femoral artery (with continuous monitoring of the oxygenation of the extremities of the lower limbs) is recommended. For differential hypoxia refractory to initial interventions, i.e., reduction in output, reduction in afterload and increase in inotropism, the conversion to V-AV ECLS or VV ECLS should be considered, based on native cardiac function.^(^167^)^ The use of left ventricular decompression measures (for example, percutaneous pulmonary artery venting) and the combination of microaxial pumps (for example, Impella™ in a configuration called ECMPELLA) should be individualized based on the hemodynamic profile.^(^167^)^

The indications, as well as contraindications, for referral of critically ill patients with cardiogenic shock associated with COVID-19 for extracorporeal cardiorespiratory support are summarized in table 5 (Appendix 1). In the therapeutic approach, ECLS should only be considered when conventional therapy fails. Prior to referral, echocardiography should be performed to assess cardiac structure and function, including biventricular function and vascular filling.

As COVID-19 is a very recent disease and ECLS is an organ support therapy used only in extremely severe cases, experience with the use of this technique in this particular context is limited and preliminary. The use of ECLS therapy should always be considered taking into account the available resources resulting from the pandemic context and the potential benefits of the support relative to the associated risks.^(^174^)^

Technological advances have made it possible to achieve excellent clinical results with ECLS in several centers worldwide, but international guidelines recommend its use in specialized centers because there is a direct correlation between the volume of ECLS cases and hospital survival.^(^178^)^ In Portugal, there are reference centers recognized by the Ministry of Health and the DGS, and interhospital transfer should be preceded whenever possible by the on-site implementation of ECLS by a dedicated rescue team to minimize the risk of clinical deterioration associated with transport.^(^174^)^

### Other organ support

**Table:** 

A conservative fluid therapy strategy **is recommended** for critically ill patients with COVID-19, especially in the absence of shock.
It **is recommended** that septic shock in critically ill patients with COVID-19 be treated based on the clinical guidelines applicable to patients with septic shock not associated with COVID-19.
It **is recommended** that nonpulmonary organ dysfunction in critically ill patients with COVID-19 be managed based on the clinical guidelines applicable to non-COVID-19 patients.

There is no direct evidence (e.g., based on specific studies) for an ideal hemodynamic support strategy for COVID-19, but it is recognized that the presence of shock, operationally defined as the need for vasopressors for mean arterial pressure (MAP) ≥ 65mmHg and lactate > 2mmol/L, in the absence of hypovolemia,^(^179^)^ in the context of SARS-CoV-2 infection is reduced (< 5%), even in intensive care patients.^(^180^)^ This fact, associated with the high risk of death from hypoxemic respiratory failure,^(^181^)^ potentially aggravated by the administration of fluids,^(^182^)^ supports the use of a conservative fluid therapy strategy, especially in the absence of tissue hypoperfusion.

In the presence of hypotension with tissue hypoperfusion, evaluated by clinical perfusion parameters (for example, capillary reperfusion time and skin temperature) and analytical parameters (for example, serum lactate), the approach is similar to that for hypotension associated with sepsis in the non-COVID-19 context.^(^183^)^ The administration of repeated boluses begins^(^98^)^ with 250 to 500cc crystalloid, ideally, balanced solutions, such as lactated Ringer’s solution or Plasma-Lyte*®,*^(^184^)^ avoiding synthetic colloids (starches, dextrans and gelatins), which are not cost effective.^(^185^)^ Albumin (20%) is as safe and effective as crystalloids but has a higher cost^(^98^,^185^)^ and should be reserved for very particular situations, such as hypoalbuminemic and hyponcotic septic patients with associated ARDS. In an intensive care setting, an echocardiogram should be performed as soon as possible, allowing a better characterization of the hemodynamic changes due to shock and facilitating the selection of the best therapeutic options, in addition to establishing a strategy for the evaluation of dynamic parameters of fluid response and guiding fluid therapy, such as variations in systolic volume, variations in pulse pressure and changes in systolic volume with a fluid challenge or, ideally, with passive elevation of the legs.^(^186^,^187^)^ This last test is performed by measuring cardiac output (by means of echocardiographic, minimally invasive or invasive methods) with the patient in a semidorsal position (head elevated 45°), positioning the patient in dorsal decubitus, with passive elevation of the patient’s lower limbs (at 45°), repeating the cardiac output measurement. This maneuver mobilizes approximately 150 - 300cc of blood from the lower body to the central circulation, resulting in an increase in preload (reversible in less than 30 seconds) and representing an increase of > 12% in cardiac output and the ability to respond to fluids.^(^187^)^

Early vasopressor perfusion may be considered for patients with severe hypotension (MAP < 50mmHg) or without tension response to the first bolus of fluid.^(^188^)^ Norepinephrine is the vasopressor of choice (which can be administered peripherally in an initial phase) and should be started at 0.5µg/minute and titrated up to 15µg/minute. Dopamine is associated with a higher incidence of arrhythmic events and mortality and should be avoided.^(^188^)^ MAP ≥ 65mmHg is considered sufficient for most patients, but patients with a history of hypertension may benefit (reduced incidence of acute kidney injury) from higher values (MAP 75 - 85mmHg) but with a higher risk of dysrhythmias.^(^189^)^ If the echocardiographic evaluation indicates changes in cardiac function associated with low/inadequate cardiac output, an inotropic agent should be administered, of which dobutamine (up to 20µg/kg/minute) is the first option.^(^98^,^190^)^ If there is an additional need to increase blood pressure, the combination or potential replacement of noradrenaline with adrenaline should be considered.^(^188^)^

The use of low doses of hydrocortisone intravenously (ideally, 200 mg per day in continuous infusion or, optionally, in a 50mg bolus every 6 hours) should be considered exclusively for patients with septic shock without response to vasopressors^(^191^)^ (operational definition, need for noradrenaline > 0.25µg/kg/minute or adrenaline > 0.25µg/kg/minute to maintain MAP within the target values). The duration of corticosteroid therapy instituted in the context of hemodynamic instability is a clinical decision that should be weighed with the need for corticosteroid therapy for other reasons in the context of COVID-19 respiratory failure.^(^98^)^

The therapeutic targets of fluid administration, associated or not with noradrenaline perfusion, are the restoration of perfusion pressure and improvements in tissue hypoperfusion, which can be evaluated by clinical signs^(^192^)^ and/or biochemical tests. The normalization of lactate (or an improvement ≥ 20%, every 2 hours, in the first 8 hours) is an appropriate therapeutic target.^(^193^)^ The use of adrenaline results in the production of aerobic lactate (through the stimulation of beta 2 adrenergic receptors in skeletal muscle), preventing the use of lactate washout to guide resuscitation.^(^188^)^

There is no direct evidence for an ideal strategy for other forms of organ support in COVID-19, but **renal support techniques** deserve special reference in the context of acute kidney injury associated with COVID-19. Before initiating renal support techniques, the reversible factors of acute kidney injury (especially prenatal causes) should be corrected,^(^194^)^ and exposure to risk factors should always be avoided (for example, administration of intravenous contrast to perform imaging tests).^(^194^)^

The indications are similar to those for non-COVID-19 critically ill patients, and outside conventional indications (severe metabolic acidemia, pH < 7.1; electrolyte changes, especially kalemia > 6.5mEq/L associated with electrocardiographic changes; drug poisoning/life-threatening dialysable toxins; overload, refractory water overload, and uremia, such as, pericarditis or encephalopathy), a delayed initiation strategy for renal support should be favored.^(^195^)^ In particular, sodium bicarbonate is known to be safe to administer (in a controlled manner) to patients with metabolic acidemia, especially of uremic etiology.^(^196^)^

Multiple renal support techniques are available, including intermittent hemodialysis (HDI), continuous renal replacement therapies (CRRTs) and hybrid therapies, also known as prolonged intermittent renal replacement therapies (PIRRTs), such as sustained low-efficiency dialysis (SLED) dialysis. There are no studies that demonstrate the practical superiority of any of the modalities, and recommendations are motivated by the need to optimize patient therapy and minimize the risk of transmission to health professionals. Thus, CRRTs are considered the preferred modality because they allow the optimization of drug dosage and the flow of dialysate to waste bags (not to the hospital sewage system) and minimize interactions with the nursing team.^(^197^)^ However, in situations where equipment isis a limiting factor, hybrid or intermittent techniques, which allow the maximization of resources, should be performed.

### Coinfection, superinfection and antimicrobials

**Table:** 

In critically ill patients with suspected severe pneumonia combined with seasonal influenza, it **is recommended** to start antibiotic therapy for influenza and reassess the clinical picture after obtaining cultural and laboratory results.
In critically ill patients with suspected severe pneumonia combined with seasonal influenza, it **is recommended** to start antibiotic therapy for influenza and reassess the clinical picture after obtaining cultural and laboratory results.
In critically ill patients with **COVID**-19, in the presence of septic shock, it **is recommended** to administer antibiotic therapy until obtaining cultural results that allow the affirmation or exclusion of the coexistence of bacterial infection.
It **is recommended** to reassess decisions regarding antibiotic therapy initiated at admission up to 72 hours, depending on the microbiological results available, the clinical evolution and inflammatory biomarkers (namely, procalcitonin).
For critically ill patients with COVID-19, it **is recommended** to maintain a high index of suspicion for nosocomial infection (namely, ventilator-associated pneumonia).
In critical patients with COVID-19 without a microbiological diagnosis or with unfavorable progression under appropriate antibiotic therapy, it **is suggested** to consider invasive pulmonary aspergillosis associated with COVID-19.

It is important to distinguish between coinfection, i.e., infection present at admission, and overinfection, i.e., infection that appears more than 48 hours after admission.

In the context of COVID-19, coinfection by other agents, even in critically ill patients, is infrequent.^(^198^,^199^)^ However, coinfection is difficult to exclude quickly, and the delay in the institution of appropriate antibiotic therapy in septic shock may be associated with increased mortality. A more liberal antibiotic therapy strategy is recommended and should be reviewed as a function of the microbiological findings, clinical evolution and inflammatory markers (namely, procalcitonin).^(^200^,^201^)^

In the context of critical COVID-19, nosocomial overinfection by other agents, particularly ventilator-associated pneumonia, is frequent.^(^202^,^203^)^ The etiological agents do not seem to differ significantly when compared to those observed in other populations, with a predominance of gram-negative bacteria (*Enterobacteria* and nonfermenters), with gram-positive bacteria present in 10% to 30% of cases.^(^202^,^203^)^ In immunocompromised patients with chronic obstructive pulmonary disease or unfavorable evolution, despite adequate antibiotic therapy, pulmonary aspergillosis associated with COVID-19 should be considered.^(^204^,^205^)^

## SPECIFIC THERAPY

### Antiviral drugs

#### Remdesivir

**Table:** 

In critically ill patients infected with SARS-CoV-2 who require a noninvasive ventilatory strategy (NIV or HFNC therapy), invasive ventilatory support, extracorporeal respiratory support or vasopressors **are recommended**; remdesivir **is not recommended**.
In critical patients infected with SARS-CoV-2 who require conventional oxygen therapy, it **is suggested** to consider the use of remdesivir in the first 72 hours after the first positive SARS-CoV-2 test.
It **is suggested** that in patients infected with SARS-CoV-2 previously treated with remdesivir with clinical deterioration, requiring escalation of ventilatory support and corticosteroid therapy, remdesivir should be maintained until the completion of the therapeutic course.

Remdesivir is an analog of adenosine that targets RNA-dependent RNA polymerase and was initially developed for the treatment of Ebola and Marburg viruses; however, it has been shown to have a spectrum of activity against other viruses.^(^206^)^ Remdesivir demonstrated in vitro efficacy in the inhibition of SARS-CoV-2, MERS-CoV and SARS-CoV-1,^(^207^)^ and in animal models infected with SARS-CoV-2, it demonstrated therapeutic activity (ability to reduce viral loads, pulmonary pathological changes and progression of clinical disease) when started early.^(^208^)^

The dose studied for the treatment of SARS-CoV-2 infection is 200mg on day 1, followed by 100 mg per day, administered intravenously (in 30 to 60 minutes), for up to 10 days. The most frequent adverse effects are gastrointestinal symptoms (nausea and vomiting), injection site reactions (phlebitis) and an increase in transaminases.

In ACTT-1 (Adaptive COVID-19 Treatment Trial), a multinational controlled and randomized study that randomized patients in the first 72 hours after a positive SARS-CoV-2 test for treatment with remdesivir or placebo, remdesivir was associated with a shorter recovery time (7 days versus 9 days) in a subgroup of patients who also received conventional oxygen therapy, at the time of randomization, with reduced progression (17% versus 24%) to noninvasive ventilation strategy (VIV or HFNC), invasive ventilatory support or extracorporeal respiratory support.^(^209^)^ The SIMPLE study compared 5 and 10 days of remdesivir treatment in patients with SARS-CoV-2 pneumonia without the need for mechanical ventilation, showing overlap between the two groups, i.e., in nonventilated patients, a 5-day therapeutic course with remdesivir is possible (no differences in mortality or adverse effects).^(^210^)^

In the SOLIDARITY trial, there was a trend toward higher mortality in critically ill patients infected with SARS-CoV-2 treated with remdesivir requiring invasive ventilatory support.^(^211^)^ A meta-analysis of multiple studies^(^209^-^212^)^ published together with the SOLIDARITY results^(^211^)^ does not allow us to conclude that remdesivir provides significant benefits. In contrast, a meta-analysis published together with a review of the guidelines of the Surviving Sepsis Campaign^(^213^)^ suggests that remdesivir may reduce the recovery time and severe adverse events (compared to standard therapy).

The Food and Drug Administration (FDA) and European Medicines Agency (EMA) approved remdesivir for patients with SARS-CoV-2 pneumonia who require supplemental oxygen therapy. However, discordant results have led the WHO to report an absence of clinical benefits with remdesivir^(^76^)^, while the Surviving Sepsis Campaign recommends its use in critically ill patients who do not require invasive ventilatory support.^(^213^)^

Considering the moderate quality evidence of clinical benefits (reduction in disease duration combined with fewer adverse events) and the potential reduction in viral clearance resulting from the use of corticosteroids, remdesivir can be considered in the first 72 hours after the first positive test for SARS-CoV-2 (inclusion criteria in ACTT-1) for patients receiving conventional oxygen therapy but not a noninvasive ventilation strategy (NIV or HFNC) or invasive ventilatory support.

The combination of an antiviral with corticosteroid therapy for some viral infections can prevent a reduction in viral clearance resulting from the use of corticosteroids.^(^214^)^ For SARS-CoV-2, there are divergent observational studies on the effect of corticosteroids on viral clearance;^(^215^,^216^)^ as such, specific studies are needed on this issue. Thus, if remdesivir has already been previously prescribed (respecting previous indications), it is suggested to complete the therapeutic course.

### Other

**Table:** 

In critically ill patients infected with SARS-CoV-2, the nonroutine use of other antivirals outside the scope of clinical use protocols or clinical trials **is recommended**.

**Lopinavir/ritonavir** is a combination of protease inhibitors used in the treatment of human immunodeficiency virus (HIV) infection; lopinavir has antiretroviral action, and ritonavir (in low dose, acts as a CYP3A inhibitor) serves as a booster of the former. Evidence from multiple randomized clinical trials, including data from the RECOVERY (Randomized Evaluation of COVID-19 Therapy) and SOLIDARITY trials, indicates that lopinavir-ritonavir is not more effective than the standard therapy for the treatment of patients with mild to severe COVID-19.^(^211^,^217^-^219^)^ Additionally, the lopinavir/ritonavir arm of the SOLIDARITY trial was discontinued due to an unfavorable adverse effects profile (in particular, gastrointestinal effects).^(^217^)^

**Darunavir**, in combination with ritonavir or cobicistat, has a mechanism of action that overlaps that of lopinavir/ritonavir, but the available evidence does not support its use in patients infected with SARS-CoV-2 because of the lack of clinical benefits and possible association with adverse events.^(^220^)^

**Favipiravir** is a broad-spectrum antiviral that targets RNA-dependent RNA polymerase; large-scale production was limited because it has a teratogenic effect.^(^221^)^ Evidence from multiple randomized clinical trials does not support its use in patients infected with SARS-CoV-2 infection because of the lack of clinical benefits associated with a not fully characterized safety profile.^(^222^-^224^)^

**Ribavirin** was tested together with lopinavir/ritonavir in patients with SARS-CoV-1,^(^225^,^226^)^ but the doses required for optimization of antiviral activity exceed the toxicity limit.

Regarding **other antivirals that act on influenza viruses (oseltamivir, umifenovir and baloxavir)**, there is no evidence available to support their use in patient with COVID-19.^(^224^)^

**Chloroquine** and its metabolite, **hydroxychloroquine**, are used as antimalarials and immunomodulators (for example, in systemic lupus erythematosus). Hydroxychloroquine and chloroquine demonstrated in vitro efficacy in the inhibition of SARS-CoV-1 and SARS-CoV-2.^(^207^,^227^)^ Initial studies on SARS-CoV-2 infection, demonstrating apparent efficacy (reduction in viral shedding time and duration of symptoms as well as an attenuation of clinical and imaging manifestations) and a good safety profile,^(^228^,^229^)^ led to an official declaration of hydroxychloroquine as a therapeutic agent for COVID-19 in China.^(^230^)^ However, evidence from multiple randomized and controlled studies, including the RECOVERY trial, showed no benefits (duration of mechanical ventilation or mortality) of antimalarials with or without azithromycin.^(^211^,^231^-^244^)^ The lack of clinical efficacy associated with the potential risk of cardiac complications (dysrhythmias, most frequently associated with QTc prolongation) led to the discontinuation by the WHO of the hydroxychloroquine arm of SOLIDARITY.^(^211^)^ A recent meta-analysis associated the use of these drugs in the context of COVID-19 with increased mortality.^(^245^)^ In Portugal, INFARMED and DGS recommended the suspension of the use of hydroxychloroquine/chloroquine in patients infected with SARS-CoV-2.^(^246^)^

**Ivermectin** is a semisynthetic drug used as an anthelmintic agent. The drug showed in vitro efficacy in the inhibition of SARS-CoV-2.^(^247^)^ The evidence available for its clinical use in SARS-CoV-2 infection comes from a meta-analysis of trials with important methodological limitations^(^248^)^ and a randomized controlled trial that indicated no benefit for its use.^(^249^)^

## IMMUNOMODULATORS

### Corticosteroids and interleukin-6 receptor inhibitors

**Table:** 

It **is recommended** that patients with COVID-19 who do not require oxygen therapy or ventilatory support should not be treated with corticosteroids unless indicated for other reasons (for example, previous therapy, acute asthma, exacerbation of chronic obstructive pulmonary disease or septic shock without response to vasopressors).
It **is recommended** that patients with COVID-19 who require oxygen therapy or ventilatory support (invasive mechanical ventilation, noninvasive mechanical ventilation or high-flow oxygen therapy by nasal cannula with a flow greater than 30 L/minute and FiO_2_ > 0.40) and are beyond 7 days since the onset of symptoms should be treated with dexamethasone 6 mg per day intravenously or enterically for up to 10 days.
It **is suggested** that for previous indications, if dexamethasone is not available, hydrocortisone (50 mg every 6 hours, intravenously), methylprednisolone (32mg daily, intravenously) or prednisolone (40 mg daily, intravenously or enterally) should be administered.
It **is suggested** that patients with COVID-19 with C-reactive protein ≥ 7.5mg/dL, ventilatory support (invasive mechanical ventilation, noninvasive mechanical ventilation or high-flow oxygen therapy by nasal cannula with a flow greater than 30L/minute and FiO_2_ > 0.40) and clinical deterioration (escalation of ventilatory support and/or worsening of PaO_2_/FiO_2_), despite corticosteroid therapy, should be treated with 8mg/kg tocilizumab (up to a maximum of 800mg) intravenously (taken only) in the first 24 hours after the start of support (must be < 14 days of hospitalization), once contraindications and other causes of deterioration of respiratory failure are excluded (for example, bacterial infection, pulmonary thromboembolism, and heart failure).
It **is suggested** that in for previous indications, if tocilizumab is not available, sarilumab (400mg) should be administered intravenously (single dose).
It **is suggested** that patients with COVID-19 receiving ventilatory support (invasive mechanical ventilation, noninvasive mechanical ventilation or high-flow oxygen therapy by nasal cannula with a flow greater than 30 L/minute and FiO_2_ > 0.40) with moderate to severe ARDS (PaO_2_/FiO_2_ < 200) and contraindications for tocilizumab should be considered for other corticotherapy protocols.

**Figure 4:**
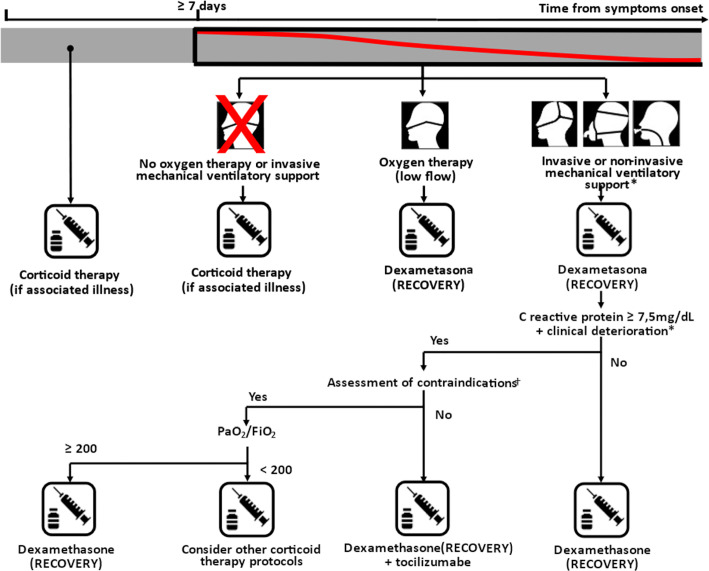
Use of corticosteroids and other immunomodulators in critically ill patients with COVID-19.

The current view on the use of corticosteroid therapy and other immunomodulators in critically ill patients with COVID-19 is summarized in figure 4 (Appendix 1).

**Corticosteroids** have antiinflammatory and antifibrotic properties that potentially accelerate the resolution of pulmonary and systemic inflammatory manifestations.^(^250^)^ This effect is beneficial in some patients with pulmonary infections (for example, pneumonia to *Pneumocystis jirovecii*)^(^251^)^ but deleterious or neutral in others (for example, flu).^(^252^)^ There is indirect evidence^(^250^,^253^)^ of the benefits (mortality and duration of mechanical ventilation) of corticosteroids in patients with ARDS (unrelated to COVID-19). A recent systematic review suggests - with a very low level of evidence - that corticosteroids can reduce mortality at 3 months and increase ventilation-free days; however, there is no evidence of an effect on mortality beyond 3 months.^(^253^)^

Initial studies pointing to prolonged viral shedding in patients infected with SARS-CoV-1 and MERS-CoV^(^214^,^254^)^ were questioned after the publication of several studies demonstrating that corticotherapy not only does not delay viral clearance^(^255^)^ but is also associated with improved clinical outcomes in patients with epidemic coronavirus infections, including SARS-CoV-2^(^45^,^256^,^257^)^ SARS-CoV-2 seems to have an earlier peak of viral replication than other viruses that cause respiratory disease, namely, SARS-CoV-1.^(^255^)^

In patients with COVID-19, the results of the corticosteroid arm of the RECOVERY trial indicate that, compared to placebo, the administration of dexamethasone (6mg per day, intravenously or enterically, for up to 10 days) improved mortality at 28 days in a subgroup of patients who required oxygen therapy, ventilatory support or extracorporeal support.^(^258^)^ In this study, the benefit was more evident in patients treated seven or more days after the onset of symptoms. In addition, a trend towards an increase in mortality was observed in patients who did not require oxygen therapy or other forms of support who received corticosteroids. These two observations support the approach that corticosteroids are indicated only when the disease is in the hyperinflammatory phase; before that, its use - unless indicated for other reasons, such as previous therapy, acute asthma, exacerbation of lung disease, obstructive or septic shock, and septic shock without response to vasopressors (operational definition, noradrenaline > 0.25µg/kg/minute or adrenaline > 0.25µg/kg/minute to maintain MAP within the target values - is potentially deleterious.

The use of dexamethasone has advantages over other corticosteroids in patients with COVID-19. It has a long half-life (up to 48 hours), allowing self-weaning; low mineralocorticoid activity, which limits hypernatremia and water retention; and good penetration into the lungs and central nervous system.^(^250^)^ Other corticosteroids, in various formulations and doses and for variable durations, were tested in patients with COVID-19 in several smaller randomized controlled studies.^(^259^-^263^)^ Many of these studies were discontinued early due to insufficient recruitment after the results of the RECOVERY trial were made available. Given that the sample size of many of these trials was insufficient to evaluate efficacy, the evidence to support the use of other corticosteroids is not as robust as that existing for dexamethasone.^(^263^)^

**Tocilizumab** is a recombinant humanized monoclonal antibody that blocks the IL-6 receptor and is used in the treatment of rheumatoid arthritis and cytokine release syndrome after therapy with T lymphocytes.^(^264^)^ Initial studies with tocilizumab did not demonstrate efficacy^(^265^,^266^)^ but were limited by low statistical power associated with heterogeneous study populations, with varying degrees of disease severity and, in particular, low use of corticosteroids (4% to 10%). In all these studies, the use of tocilizumab was considered safe, and although neutropenia occurred, there was not an increase in the rate of infection with clinical expression. Subsequent studies have demonstrated safety and efficacy. The COVACTA (A Study to Evaluate the Safety and Efficacy of Tocilizumab in Patients with Severe COVID-19 Pneumonia) study demonstrated a reduction in the incidence or duration of hospitalization and admission to intensive care,^(^267^)^ and the EMPACTA (Evaluating Minority Patients With Actemra) study showed a reduced incidence of the need for mechanical ventilation and of death.^(^268^)^ Most patients included in COVACTA were receiving ventilatory support and, in EMPACTA, receiving corticotherapy, suggesting that these factors alone or in combination may contribute to the differences in the therapeutic effect of tocilizumab.

The tocilizumab arms of the REMAP-CAP (Randomized Embedding Multifactorial Adaptive Platform for Community-acquired Pneumonia) trial^(^269^)^ and RECOVERY^(^270^)^ demonstrated, in selected populations of patients with COVID-19, a benefit of the drug with regard to mortality. The tocilizumab arm of REMAP-CAP recruited exclusively critically ill patients with COVID-19, requiring ventilatory support, in the first 24 hours of admission to intensive care and in the first days of hospitalization, with the majority (> 90%) undergoing concomitant corticosteroid therapy. REMAP-CAP showed a reduction in mortality as well as in the length of hospital stay and an increase in the number of days without organ support.^(^269^)^ In the RECOVERY trial, a subgroup of patients hospitalized with COVID-19 with hypoxemia (SpO_2_ < 92% or need for supplemental oxygen) and a C-reactive protein concentration ≥ 7.5mg/dL was randomized for the administration of tocilizumab (versus placebo), with the majority (> 80%) receiving concomitant corticosteroid therapy and more than half receiving ventilatory support. RECOVERY showed a reduction in mortality as well as in the length of hospital stay, but this mortality benefit was restricted to patients receiving concomitant corticosteroid therapy.^(^270^)^

Some patients who receive conventional oxygen therapy, i.e., without the need for ventilatory support, with significant systemic inflammation and progressive hypoxemia, may benefit from the addition of tocilizumab to standard therapy, but there is currently insufficient evidence to define this subgroup of patients. Thus, considering the scarcity of IL-6 receptor blockers, this therapy should be prioritized for patients with greater need and greater probability of benefiting from the therapy.

There are different dosing schedules recommended for COVID-19, with the greatest concensus for 8mg/kg body weight (up to a maximum dose of 800mg) intravenously (slow perfusion). Some protocols recommend repeated administration after 12 hours if the response is incomplete (a maximum of two doses).

The use of tocilizumab should be avoided if there is significant immunosuppression, particularly in patients using other immunomodulatory biological drugs; in patients with alanine transaminase > 5 times the upper limit of normal; in patients with a high risk of gastrointestinal perforation (for example, diverticulitis); in patients with uncontrolled bacterial, fungal or viral infection (non-SARS-CoV-2); and in patients with an absolute neutrophil count < 500 cells/µL or platelet count < 50,000 cells/µL.

C-reactive protein is directly inhibited by IL-6 blockade and thus cannot be used as a marker for suspected concomitant infection or for monitoring the response to antimicrobial therapy; instead, procalcitonin should be used. The half-life of the drug is long, and its effect lasts, in most circumstances, at least three weeks.^(^264^)^

**Sarilumab,** a direct inhibitor of IL-6, is a human monoclonal antibody used in the treatment of rheumatoid arthritis. The evidence regarding the efficacy of sarilumab in critically ill patients with COVID-19 comes from the REMAP-CAP trial, and the data are less robust than those for tocilizumab (less than 50 patients were included in the study),^(^269^)^ making it an option only when the former is unavailable. The recommended dose regimen is 400mg intravenously (single dose).

The Surviving Sepsis Campaign guidelines for COVID-19^(^98^)^ were updated based on a recent Cochrane review^(^253^)^ and the DEXA-ARDS study,^(^250^)^ which showed reductions in both mortality and duration of mechanical ventilation in patients with moderate to severe ARDS; however, these results should be applied with caution to COVID-19 because they include patients with nonviral ARDS. Other corticosteroid protocols (Table 6-Appendix 1) have a lower degree of evidence with regard to COVID-19, but sarilumab should only be considered for patients with severe forms of respiratory failure in the context of SARS-CoV-2 infection when there are formal contraindications to the previously described approaches and when the risk-benefit ratio may be more favorable.

### Other immunomodulators

**Table:** 

In critically ill patients infected with SARS-CoV-2, the nonroutine use of other antivirals outside the scope of clinical use protocols or clinical trials **is recommended**.

**Anakinra** is a recombinant protein that acts as an IL-1 receptor antagonist, is used in the treatment of rheumatoid arthritis and autoinflammatory syndromes and is considered one of the safest immunomodulators (rarely associated with opportunistic infections).^(^271^)^ In two observational studies in patients with severe COVID-19 (under NIV with PaO_2_/FiO_2_ < 200) in the hyperinflammatory phase (C-reactive protein > 10mg/dL and/or ferritin > 900ng/mL, after exclusion of bacterial infection), anakinra therapy with a high-dose protocol (5mg/kg twice a day, intravenously) was associated with sustained respiratory improvement and a reduction in admission to intensive care.^(^272^,^273^)^ The CORIMUNO-ANA-1 randomized trial, which included patients with mild to moderate COVID-19, concluded that anakinra can reduce mortality and the need for invasive mechanical ventilation (or ECLS) without significant adverse effects. However, the degree of evidence is low, given the lack of blinding and the wide confidence intervals for mortality and other endpoints.^(^274^)^

**Baricitinib** is a reversible JAK (*Janus kinase*) 1 inhibitor approved for the treatment of rheumatoid arthritis and, in the context of COVID-19, caused a reduction in all-cause mortality and time to symptom resolution (associated with a better adverse effects profile), especially in patients undergoing NIV and HFNC therapy.^(^275^)^ There are not yet enough data to validate this therapy in critically ill patients.

**Colchicine** is a drug that inhibits the polymerization of mitotic spindle proteins (i.e., stops cell division in metaphase) and is used as an antiinflammatory agent in the treatment of gout, pericarditis, inflammatory arthritis, familial Mediterranean fever and Behçet’s disease. The drug was studied in different clinical trials that cumulatively did not demonstrate efficacy with regard to mortality and other relevant endpoints but in which there was an increased incidence of adverse events, especially gastrointestinal events.^(^276^-^278^)^

**Interferons** (IFNs), of which there are three classes, type I (IFN-α and IFN-β), type II (IFN-γ) and type III, are a group of cytokines capable of inducing an antiviral-resistant state in noninfected tissue cells,^(^279^)^ and SARS-CoV-2 is known to suppress the production of type I IFNs.^(^280^)^ Although some studies, with obvious methodological limitations, have demonstrated the efficacy of IFN-β,^(^281^)^ the results were not confirmed by the provisional SOLIDARITY results.^(^211^)^ **Inhaled IFN-**β, an experimental formulation of the drug administered by nebulization, was evaluated in a randomized study in noncritical patients and was associated with a lower risk of progression to severe disease but without a significant impact on mortality.^(^282^)^

### Anticoagulation

**Table:** 

It **is recommended** that critically ill patients with COVID-19 with confirmation (or high clinical suspicion) of thromboembolic disease receive therapeutic strategies, including reperfusion (pharmacological and/or mechanical) and/or therapeutic anticoagulation regimens following standard institutional protocols.
It **is recommended** that critically ill patients with COVID-19, previously under a therapeutic anticoagulation regimen at home, maintain a therapeutic anticoagulation regimen. A transition from parenteral anticoagulant agents (for example, low molecular weight heparin or unfractionated heparin) to oral anticoagulants (for example, dicoumarin or new oral anticoagulants) is suggested.
It **is recommended** that critically ill patients with COVID-19 without evidence of thromboembolic disease should be medicated with a prophylactic anticoagulation regimen (standard or adjusted) in the absence of contraindications.
It **is recommended** that critically ill patients with COVID-19 receiving extracorporeal organ support (including veno-venous or veno-arterial extracorporeal life support and renal support therapy) receive antithrombotic therapy following standard institutional protocols.

Critically ill patients with confirmed or high clinical suspicion of COVID-19 (for example, ventilatory deterioration and/or sudden hemodynamic instability, especially in the presence of right ventricular dysfunction, in the context of pulmonary thromboembolism) and thromboembolic disease should receive therapeutic strategies that include reperfusion (pharmacological and/or mechanical) and/or therapeutic anticoagulation regimens following standard institutional protocols.^(^283^)^ Critically ill patients with COVID-19 receiving extracorporeal organ support (including veno-venous or veno-arterial extracorporeal life support and renal support therapy) should receive antithrombotic therapy following the established institutional protocols.

Other than these classic indications, there is no evidence of benefits of the preemptive use of a therapeutic anticoagulation regimen, and at least one observational study showed an increased risk of in-hospital mortality (2.3-fold increase in mortality), even in patients with higher inflammatory activity (increased C-reactive protein ≥ 20 mg/dL^(^284^)^).

**Figure 5:**
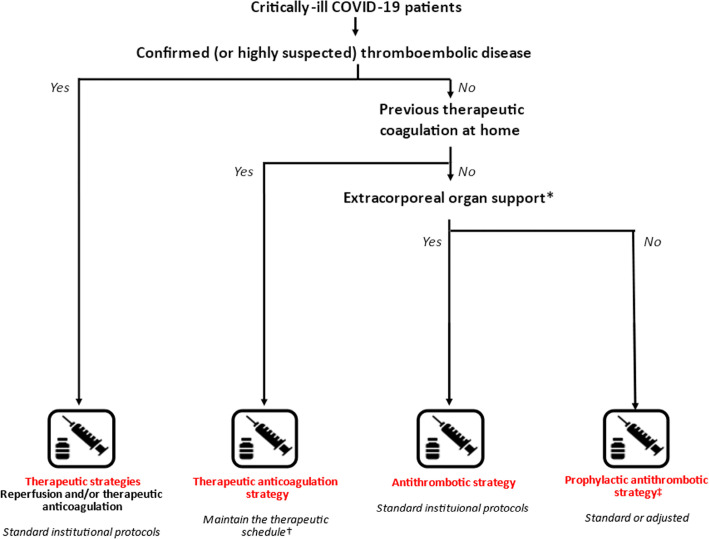
Recommendations for the use of different anticoagulation regimens for critically ill patients with COVID-19.

Figure 5 (Appendix 1) illustrates the recommendations for the use of different anticoagulation regimens for critically ill patients with COVID-19.

A prepublication^(^285^)^ of a multiformat international study was recently made available; the study evaluated data from three randomized and independent controlled trials (REMAP-CAP, ACTIV-4 (Therapeutic Anticoagulation, Accelerating COVID-19 Therapeutic Interventions and Vaccines-4) and ATTACC (Antithrombotics Inpatient and Antithrombotic Therapy to Ameliorate Complications of COVID-19) and compared the efficacy of therapeutic and prophylactic anticoagulation regimens in hospitalized patients who did and did not require organ support (defined as vasopressor inotropic support, high-flow nasal oxygen therapy, invasive or noninvasive mechanical ventilation, or ECLS). After provisional analysis, the recruitment of patients was interrupted for the group of hospitalized patients who required organ support because of futility in relation to the primary objective (reduction in the need for organ support at 21 days) and a possible increased risk of bleeding (increased absolute number of patients with major hemorrhagic events) with the therapeutic anticoagulation regimen (in relation to the prophylactic regimen). These results are different from those for the group of hospitalized patients who did not require organ support, in which recruitment was also interrupted but because of the superiority of the therapeutic anticoagulation regimen (in relation to the prophylactic regimen) with regard to the primary objective.

Thus, the current evidence points to a prophylactic anticoagulation regimen as the primary anticoagulation strategy in critically ill patients (in need of organ support) in the absence of modifying situations or contraindications,^(^286^)^ especially the presence of active bleeding or thrombocytopenia (with a platelet count less than 25,000/µL).

This strategy is supported by all international organizations (Anticoagulation Forum, American College of Chest Physicians, International Society on Thrombosis and Hemostasis, Italian Society on Thrombosis and Hemostasis, North American Thrombosis Forum, European Society of Vascular Medicine and International Union of Angiology) who endorse such clinical guidelines.^(^287^-^291^)^ In these standards, heparins (low molecular weight or unfractionated) are the anticoagulants of choice, even in patients undergoing home anticoagulation therapy with other agents,^(^292^)^ for the history of their use in intensive care but also for their pleotropic effects, especially their antiinflammatory activity.^(^293^)^ However, the dosage for the prophylactic anticoagulation regimen is controversial. In critically ill patients without COVID-19, there is a growing body of evidence that demonstrates that the doses commonly used in prophylactic anticoagulation regimens are inadequate and that higher doses are necessary.^(^294^,^295^)^ Some international standards recommend the use of higher doses in critically ill patients with COVID-19^(^287^,^290^)^ and adjustments to the formulation/dose based on weight, in accordance with the guidelines considered in other scenarios,^(^296^)^ with possible monitoring of anti-Xa activity to reduce the bleeding risk^(^292^)^ and/or renal function.^(^292^)^ In the prepublication that analyzes REMAP-CAP, ACTIV-4 and ATTACC,^(^285^)^ 51.3% of patients included in the prophylactic regimen group used intermediate doses of anticoagulant, corresponding to the adjusted doses in the prophylactic anticoagulation regimen. A recent randomized controlled trial with patients with critical COVID-19 showed no statistically significant differences between standard and adjusted prophylactic anticoagulation regimens (enoxaparin 1mg/kg per day; not the optimal dose from the pharmacokinetic point of view); thus, there is still no concensus on the choice for the ideal scheme.

Table 7 (Appendix 1) provides the different prophylactic (standard and adjusted) and therapeutic anticoagulation regimens available for critically ill patients with COVID-19.

### Other therapies

**Table:** 

In critically ill patients infected with SARS-CoV-2, it **is recommended** *not* to use convalescent plasma therapy outside the scope of clinical use protocols or clinical trials.

Therapy with **convalescent plasma** is based on the principle of passive immunity, a technique in which plasma rich in antibodies from individuals in the convalescence phase of an infectious disease is administered to others in the acute phase of the same disease to confer short-term immunity.^(^297^)^ In the specific context of COVID-19, neutralizing antibodies are those that bind to the spike protein and prevent its interaction with the ACE2 receptor or block its conformational changes, preventing fusion to the membrane of host cells.^(^298^)^ Although a recent Cochrane review^(^299^)^ revealed a high degree of uncertainty regarding the efficacy of convalescent plasma therapy, the FDA approved this therapy for critically ill patients.^(^300^)^ The evidence stems from multiple randomized clinical trials that compared convalescent plasma with standard treatment in patients with mild,^(^301^)^ moderate^(^302^-^304^)^ and severe COVID-19,^(^298^,^305^,^306^)^ who showed improvements in dyspnea but without significant differences in relation to other outcomes (mortality, need for invasive mechanical ventilation, admission to intensive care and time to hospital discharge) and at the expense of an increase in serious adverse events. In Portugal, a working group was created for the development and proposal of a National Program for Convalescent Plasma Transfusion for the Treatment of Patients with COVID-19.^(^307^)^

**Table:** 

In critical patients infected with SARS-CoV-2, it **is recommended** *not* to use therapy with mesenchymal stem cells outside the scope of clinical use protocols or clinical trials.

**Mesenchymal stem cells** isolated from various donor sites (bone marrow, placenta, fat or umbilical cord) can be administered intravenously, producing powerful and comprehensive immunomodulatory functions.^(^308^)^ The safety and efficacy of the administration of these cells, especially those from umbilical cord tissue, have been clearly documented in multiple clinical trials,^(^309^)^ especially for inflammatory diseases involving the immune system, such as graft-versus-host disease.^(^310^)^ Multiple randomized clinical trials have compared mesenchymal stem cell therapy with standard therapy for patients with mild to severe COVID-19,^(^311^-^313^)^ but the confidence for all results (mortality and duration of ventilation) was very low because of the high risk of bias and inaccuracy.

The use of mesenchymal stem cells for the treatment of patients with COVID-19 has biological plausibility, but randomized and quality-controlled studies are needed before the use of this intervention can be considered outside the scope of the clinical use protocols or clinical trials.^(^314^)^ In Portugal, a company provides technology, resources and products *pro bono* and in a timely manner. The current form of access is through an application for an Authorization for Exceptional Use (AUE) required by hospitals (after careful evaluation by the Pharmacy and Therapeutic Committee), but the inclusion of patients in a clinical trial is being considered.

**Table:** 

In critically ill patients infected with SARS-CoV-2, it **is recommended** *not* to use therapy with neutralizing antibodies outside the scope of clinical use protocols or clinical trials.

**Bamlanivimab** is an IgG1κ recombinant human monoclonal antibody that neutralizes the SARS-CoV-2 spike protein. The BLAZE-1 (outpatients with mild COVID-19) and ACTIV-3/TICO (patients with moderate to severe COVID-19) trials showed no improvement in any outcome (mortality, hospitalization, virological clearance, clinical recovery rate and adverse effects) compared to standard therapy.^(^315^,^316^)^

**REGN-COV2** is a combination of two neutralizing antibodies (casirivimab and imdevimab) against the SARS-CoV-2 spike protein. The drug is being studied in patients with mild to moderate COVID-19 (nonhospitalized), and preliminary data have not shown clinical efficacy compared to placebo.^(^317^)^

### Other drugs

**Table:** 

In critically ill patients infected with SARS-CoV-2, routine nonsuspension of chronic therapy with renin-angiotensin system inhibitors (angiotensin converting enzyme (ACE) inhibitors or angiotensin 2 receptor antagonists) or statins **is recommended**.

There are no randomized controlled studies that have analyzed the benefit of maintaining or discontinuing chronic therapy with angiotensin converting enzyme inhibitors (ACEIs) or angiotensin 2 receptor antagonists in patients infected (or with a risk of infection) with SARS-CoV-2. Multiple observational studies have demonstrated that it is unlikely that the continuous use of these drugs is associated with an increased risk of disease severity (or death) and that there is a quantifiable risk of decompensation of heart failure or worsening of blood pressure control if chronic therapy is abruptly discontinued.^(^318^-^321^)^ Thus, the *Sociedade Portuguesa de Cardiologia*,^(^322^)^ along with multiple scientific societies (e.g., American Heart Association (AHA) and American College of Cardiology (ACC)),^(^323^)^ considers that there is no clinical or scientific evidence to support the routine interruption of chronic therapy with drugs in this group for patients infected with (or with a risk of infection) with SARS-CoV-2. In the specific context of critically ill patients infected with SARS-CoV-2, the risks and benefits of therapy should be weighed in each case, considering the different comorbidities and organ dysfunctions.

Despite the concern with the hepatotoxicity of statins, mainly because an increase in transaminases is common in SARS-CoV-2 infection, the evidence points to a low risk of toxicity,^(^324^)^ and multiple scientific societies (for example, AHA and ACC)^(^323^)^ recommend the continuation of statin therapy in hospitalized patients infected with SARS-CoV-2.

**Table:** 

In critically ill patients infected with SARS-CoV-2, it **is recommended** not to discontinue or avoid treatment with nonsteroidal antiinflammatory drugs (NSAIDs) when clinically indicated.

Concern about the possible adverse effects of NSAIDs was raised by anecdotal reports of the rapid progression of some patients infected with SARS-CoV-2 who taking these drugs.^(^325^)^ In the absence of clinical or population data that substantiate this fact, EMA^(^326^)^ and the WHO^(^327^)^ do not recommend discontinuation or avoidance of NSAID therapy when clinically indicated. Thus, consistent with the general approach to fever in adults, paracetamol should be the preferred antipyretic, with NSAIDs used as second-line drugs (at the lowest effective dose).

### Criteria for cure and suspension of isolation

**Table:** 

It **is recommended** that obtaining a cure criterion (and consequent suspension of isolation) of patients with severe or critical COVID-19 (or severe immunosuppression, regardless of the severity of the disease) does not depend on laboratory criteria but rather on the cumulative fulfillment of criteria: (1) clinical (significant improvement of symptoms with apyrexia, without use of antipyretics, for three consecutive days) and (2) temporal (20 days since the onset of symptoms).

The determination of the cure criteria for SARS-CoV-2-infected individuals is essential to maximize the suspension of unnecessary isolations, with the distribution of patients to clean areas, without compromising the safety of other patients and health professionals.^(^328^)^

The presence of SARS-CoV-2 virus genetic material in a biological sample is regarded as a positive test, but such positivity does not necessarily imply that the virus is viable, i.e., transmit from person to person. Most SARS-CoV-2-infected individuals are not NAAT positive approximately 2 weeks after infection, but approximately 5%-10% of infected individuals, especially critically ill patients and those with severe immunosuppression, remain positive after this period, and occasionally, patients with previous negative tests return positive tests after a short period of time (< 3 months).^(^329^,^330^)^

The recommendations of the DGS,^(^328^)^ which are based on the guidelines issued by the WHO^(^330^)^ and by the ECDC,^(^329^)^ recommend a strategy to define a cure criterion - and consequent suspension of isolation - for patients with severe or critical COVID-19 (or severe immunosuppression, regardless of the severity of the disease) determined by clinical criteria, such as significant improvement of symptoms with apyrexia (without use of antipyretics) for three consecutive days, and temporality, such as 20 days since the onset of symptoms, without the need for laboratory criteria (NAAT negative for SARS-CoV-2).

Severe immunosuppression situations can occur in the context of active malignancy (particularly for patients undergoing chemotherapy, radiotherapy or immunotherapy/biologicals); allogeneic transplantation of hematopoietic progenitor cells within less than 1 year or graft-versus-host disease; lung transplantation or other organ transplantation within 6 months or rejection within 3 months; biological therapy and/or prednisolone-equivalent dose > 20mg/day for more than 14 days; HIV infection without therapy and with a CD4+ T cell count < 200 cells/mm^3^; and primary immunodeficiency (severe combined immunodeficiency syndrome, X-linked agammaglobulinemia, interferon receptor deficiency and hyper-IgE syndrome).^(^328^)^

## CONCLUSION

The COVID-19 pandemic is an important cause of morbidity and mortality for which scientific knowledge has grown and changed at an accelerated pace. Given the nature of the pandemic and considering the constant changes in clinical and political knowledge, it is necessary to review and summarize the scientific literature to inform and decide on best practices from an evidence-based perspective. These recommendations provide recommendations/suggestions for the organization of health services and management of patients with COVID-19 in intensive care departments, being specifically oriented to the Portuguese reality, African Countries of Portuguese Official Language and East Timor. Its need is urgent in a world of constant disinformation and change, in which certain actions have a great prognostic impact on patients. The present recommendations should be continuously reviewed to reflect advances in our understanding and treatment of this pathology, constituting a living and up-to-date document.

## Figures and Tables

SARS-CoV-2 - severe acute respiratory syndrome coronavirus 2; ACE2 - angiotensin 2 -converting enzyme; TMPRSS2 - serine protease transmembrane type 2; IL - interleukin; TNF-α - tumor necrosis factor alpha; P-SILI - patient self-inflicted lung injury.

PaO_2_ - partial pressure of oxygen; FiO2 - fraction of inspired oxygen; CRP - C-reactive protein; AST/ALT - aspartate/alanine aminotransferase; LDH - lactate dehydrogenase; TnI - troponin I; BNP - B-type natriuretic peptide; ECDs - complementary diagnostic tests; CT - computed tomography; rRT-PCR - real-time reverse transcription-polymerase chain reaction.

SpO_2_ - peripheral oxygen saturation; E respiratory - respiratory effort; HFNC - high-flow nasal cannula; TºC - temperature in degrees Celsius; FiO_2_ - fraction of inspired oxygen; CPAP - continuous positive airway pressure; BPAP - bilevel positive airway pressure; RR - respiratory rate; PaO_2_ - partial pressure of oxygen; PEEP - positive end -expiratory pressure; ECLS - extracorporeal life support.

* Clinical deterioration: (1) escalation of ventilatory support and/or (2) worsening of partial pressure of oxygen/fraction of inspired oxygen; † contraindications: (1) significant immunosuppression, particularly in patients using other immunomodulatory biological drugs; (2) alanine transaminase > 5 times the upper limit of normal; (3) high risk of gastrointestinal perforation (for example, diverticulitis); (4) uncontrolled bacterial, fungal or viral (non-SARS-CoV-2) infection; (5) absolute neutrophil count < 500 cells/µL; or (6) platelet count < 50,000 cells/µL.

* Veno-venous or veno-arterial extracorporeal life support, renal support therapy, and/or others; † transition from oral anticoagulant agents to parenteral anticoagulant agents is suggested; ‡ in the absence of contraindications - presence of active bleeding or thrombocytopenia (with platelet count < 25,000/µL).

## Appendix 1 - Figures and tables

**Table 1:** Definitions of COVID-19 severity

**Asymptomatic or presymptomatic infection**	Individuals positive for SARS-CoV-2* *without* signs/symptoms consistent with COVID-19
**Mild disease**	Individuals with a positive SARS-CoV-2* test *with* signs/symptoms consistent with COVID-19 (for example, fever, myalgia, headache, ageusia, anosmia, nausea/vomiting or diarrhea) *without* signs/symptoms involving the lower respiratory tract or radiographic alterations
**Moderate disease**	Individuals with a positive SARS-CoV-_2_* test *with* signs/symptoms involving the lower respiratory tract (for example, fever, cough, dyspnea and tachypnea) and radiographic evidence of pneumonia, *with* SpO_2_ ≥ 90% in room air, and an absence of hemodynamic instability
**Severe disease**	Individuals with a positive SARS-CoV-2* test *with* signs/symptoms involving the lower respiratory tract (for example, fever, cough, dyspnea and tachypnea) and radiographic evidence of pneumonia, *with* SpO_2_ < 90% in room air, respiratory rate > 30cpm, *or* increased respiratory effort (at least one of the criteria) and an absence of hemodynamic instability
**Critical illness**	Individuals with a positive test for SARS-CoV-2* and adult respiratory distress syndrome (ARDS)†, sepsis‡ or septic shock§, and/or an acute thrombotic or thromboembolic event (for example, embolism, stroke, or acute myocardial infarction)

SARS-CoV-2 - severe acute respiratory syndrome coronavirus 2; SpO_2_ - peripheral oxygen saturation; cpm - cycles per minute; PaO_2_/FiO_2_ - partial pressure of oxygen/fraction of inspired oxygen.

* Nucleic acid amplification test or antigen test; † meets the Berlin definition criteria:^(^331^)^ hypoxemic respiratory failure (PaO_2_/FiO_2_ ≤ 300 with positive end-expiratory pressure expiration/continuous positive airway pressure ≥ 5cmH_2_O) with acute onset <1 week after known risk factor, characterized by bilateral opacities (not explained by effusion, atelectasis or nodules) and not explained by heart failure or fluid overload (exclusion by clinical and laboratory criteria and echocardiographic evaluation); ‡ meets the Surviving Sepsis Campaign criteria:^(^183^)^ Life dysfunction and threat to organs (clinically operationalized as an acute increase of ≥ 2 points on the Sequential Organ Failure Assessment) caused by unregulated host response to infection; § meets the Surviving Sepsis Campaign criteria:^(^183^)^ subgroup of patients with sepsis (clinically identified by serum lactate > 2 points/L in the absence of hypovolemia; and § meets the Surviving Sepsis Campaign criteria ≥ 65) subgroup of particular circulatory/metabolic condition.

**Table 2:** Systematized protocol for endotracheal intubation in the context of COVID-19

(1)	Preoxygenation with a high-concentration facial mask or Mapleson C balloon system connected to a high-efficiency respiratory filter, always without the use of manual insufflation
(2)	Fast sequence intubation technique
(3)	Videolaryngoscopy with the use of a disposable blade
(4)	Postintubation with tube closure with a clamp until connected to a manual ventilator (or Mapleson C type balloon system) or mechanical ventilator equipped with a high-efficiency respiratory filter
(5)	Confirmation of intubation by capnography/capnometry followed by chest radiography (without auscultation)

**Table 3:** Systematized extubation protocol in the context of COVID-19

(1)	Aspirate bronchial, oral and pharyngeal secretions
(2)	Put ventilator in standby mode immediately before extubation
(3)	Keep the aspiration probe below the level of the tube during and after cuff deflation
(4)	Gently remove the endotracheal tube during inspiration
(5)	Discard the endotracheal tube, as well as the entire ventilatory circuit, in a biohazard bag

**Table 4:** Referral of critically ill patients with respiratory failure associated with COVID-19 for extracorporeal respiratory support

Inclusion criteria*
**Emerging criteria**
Refractory hypoxemia (PaO_2_/FiO_2_ < 50, for > 3 hours)
Circulatory shock associated with severe acute cor pulmonale
**Urgent criteria**
Severe hypoxemia (PaO_2_/FiO_2_ < 80, for > 6 hours)
Severe respiratory acidosis (pH < 7.25 with PaCO_2_ > 60mmHg, for > 6 hours)
**Nonurgent criteria**
Maintenance of non-protective ventilatory parameters †
**Exclusion criteria**
Severe respiratory failure requiring prolonged (noninvasive or invasive) ventilatory support (> 7 days)
Multiorgan failure
Uncontrolled hemorrhage
Acute brain injury
Significant comorbidity
Physiological fragility (clinical frailty scale > 3)

PaO_2_ - partial pressure of oxygen; FiO_2_ - fraction of inspired oxygen.

* After optimization of invasive mechanical ventilation; † tidal volume > 6cc/kg of ideal weight; plateau pressure > 30cmH_2_O; FiO_2_ > 60%.

**Table 5:** Referral of critically ill patients with cardiogenic shock associated with COVID-19 for extracorporeal cardiorespiratory support

Inclusion criteria
**Emerging criteria**
Refractory cardiocirculatory arrest*
**Urgent criteria**
Critical cardiogenic shock †
**Nonurgent criteria**
Progressive cardiogenic shock ‡
**Exclusion criteria§**
Significant previous heart disease
Multiorgan failure
Uncontrolled hemorrhage
Acute brain injury
Significant comorbidity
Physiological fragility (*clinical frailty scale* > 3)

* > 30 minutes of advanced life support; † hypotension refractory to increased pharmacological support complicated by severe organic hypoperfusion with increasing hyperlactacidemia; ‡ progressive hemodynamic deterioration, despite pharmacological support, associated with organ dysfunction (e.g., acute kidney injury); § In refractory cardiocirculatory arrest, the presence of previous no-flow at the beginning of resuscitation maneuvers and cardiocirculatory support by extracorporeal membrane oxygenation < 60 minutes after collapse are contraindications.

**Table 6:** Most common corticosteroid regimens in ARDS studies

Protocol DEXA-ARDS^(^250^)^
Days 1 to 5: dexamethasone (20mg/day) intravenously
Days 6 to 10: dexamethasone (10mg/day) intravenously
**CIRCI Protocol^(^332^)^**
Initial bolus with methylprednisolone (1mg/kg) intravenously (ideally in 30 minutes)
Days 1 to 14*: methylprednisolone (1mg/kg/day) intravenously †
Days 15 to 21: methylprednisolone (0.5mg/kg/day) intravenously †
Days 22 to 25: methylprednisolone (0.25mg/kg/day) intravenously †
Days 26 to 28: methylprednisolone (0.125mg/kg/day) intravenously †

* If the patient is extubated between days 1 and 14, start weaning the next day (day 15); † round to the nearest decimal place, dilute in 50cc of saline and infuse at 2.1cc/h *or* half bolus dose intravenously (ideally in 30 minutes) every 12 hours; after 5 days of tolerance to enteric diet, can administer the equivalent dose of enteric prednisolone (per os or nasogastric tube).

CIRCI - Critical Illness-Related Corticosteroid Insufficiency.

**Table 7:** Prophylactic (standard and adjusted) and therapeutic anticoagulation schemes for critically ill patients with COVID-19

	Prophylactic anticoagulation scheme		Therapeutic anticoagulation scheme
	Standard	Adjusted (intermediate dose)	
**Standard dose**	Enoxaparin (40mg) once daily subcutaneously	Enoxaparin (0.5mg/kg) twice per day subcutaneously *or* Enoxaparin (40mg) twice per day subcutaneously*	Enoxaparin (1.0mg/kg) twice per day subcutaneously
**IMC ≥ 40kg/m^2^**	Enoxaparin (40mg) twice per day subcutaneously		Enoxaparin (1.0mg/kg) twice per day subcutaneously †
**Creatinine clearance < 30mL/minute**	Unfractionated heparin (5,000IU) 3 times per day subcutaneously	Unfractionated heparin (7,500IU) 3 times per day subcutaneously	Enoxaparin (1.0mg/kg) once per day subcutaneously *or* unfractionated heparin (perfusion) intravenously

IMC - índice de massa corporal.

* Escolher o esquema que proporcione a dose superior; † monitorização de atividade de anti-Xa < 1,2UI/mL.

## Appendix 2 - Summary of Recommendations

**Table:**

**Diagnosis of SARS-CoV-2 infection**
It **is recommended** that all patients requiring hospitalization in intensive care units undergo a diagnostic test to identify SARS-CoV-2.
It **is recommended** that the initial diagnostic test in patients requiring hospitalization in intensive care units be a molecular nucleic acid amplification test (NAAT) using a sample from the upper respiratory tract (exudate from the nasopharynx and oropharynx collected with a swab) in the context of pneumonia, whenever possible, to the lower respiratory tract (for example, bronchial secretions collected by endotracheal aspirate).
It **is suggested** that when NAAT results cannot be obtained in less than 12 hours (or if NAATs are not available), a rapid antigen test should be used, and a confirmatory NAAT should be conducted as soon as possible if the rapid antigen test result is negative.
It **is suggested** that during hospitalization, between the third and fifth day after the initial negative test and periodically every 5 days (counted from the last test), NAATs should be used for screening.
It **is recommended** *not* to use serological tests in the acute phase.
It **is recommended** *not* to use chest CT as the first diagnostic test in patients with suspected SARS-CoV-2 infection.

**Table:**

**Diagnosis of co-infection and superinfection**
The collection of blood cultures (at least two sets of aerobic and anaerobic blood cultures) from the lower respiratory tract is **recommended** for the investigation of other microbiological agents and antigenuria for *Legionella pneumophila* and *Streptococcus pneumoniae*.
It **is suggested** to consider requesting other tests (for example, NAATs for other viruses, e.g., influenza, and other respiratory viruses, serology for atypical microorganisms, galactomannan detection) based clinical symptoms and epidemiology.

**Table:**

**Criteria for admission to intensive care units**
**It is recommended** that patients with severe or critical COVID-19 criteria be referred early to intensive care units.
**It is recommended** that admission to the intensive care unit be based on a case-by-case assessment that includes the presentation and severity of acute disease, the reversibility and favorable prognosis of acute disease, history of comorbidities, and poor functional status and frailty prior to the acute situation motivating admission.
**It is recommended** that whenever there is no possibility of a local response, referral and transfer of the patient should be based on the intensive care referral network so that the necessary care can be provided.
**It is recommended** that the decision to admit (or not) be accompanied by the development of a care plan based on a decision model shared with the patient or with his or her family; collegial methodology, ideally multiprofessional and multispecialty, coordinated by an experienced intensivist; and the use of national and international standards and guidelines.

**Table:**

**Personal protective equipment**
It **is recommended** that all health professionals involved in the provision of clinical care to patients with (or suspected of) infection by coronavirus severe acute respiratory syndrome 2 (SARS-CoV-2) use universal protection, contact protection and droplet protection. These measures include hand hygiene and the use of specific, disposable (single use) and waterproof personal protective equipment: surgical mask, eye protection, cap, smock, clean gloves (covering the cuff) and footwear protection (ideally, waterproof shoes and exclusive use in isolation areas or, optionally, waterproof shoe covers).
It **is recommended** that all health professionals involved in the provision of clinical care to patients with (or suspected of) infection by coronavirus severe acute respiratory syndrome 2 (SARS-CoV-2) use universal protection, contact protection and droplet protection. These measures include hand hygiene and the use of specific, disposable (single use) and waterproof personal protective equipment: surgical mask, eye protection, cap, smock, clean gloves (covering the cuff) and footwear protection (ideally, waterproof shoes and exclusive use in isolation areas or, optionally, waterproof shoe covers).
It **is recommended** that all health professionals involved in the provision of potentially aerosol-generating clinical care (for example, intubation, secretion aspiration, and bronchoscopy) or prolonged contact (> 15 minutes) and/or intimate contact (for example, placement of a central venous catheter, surgery, and cardiopulmonary resuscitation maneuvers) to patients with (or suspected of) SARS-CoV-2 infection use airway protection. These measures include hand hygiene and the use of specific, disposable (single use) and waterproof personal protective equipment: respirator with a facial filter, eye protection (with side protection), cap, smock (with cuffs that tighten or with elastics and that cover up to the middle of the leg or ankle) and apron, clean gloves (covering the cuff of the gown) and footwear protection (ideally waterproof shoes and exclusive use in isolation areas or, optionally, waterproof shoe covers).
It **is suggested** that full protection (waterproof, with built-in hood and neck protection) be limited to professionals with training and practical experience for this purpose.
It **is suggested** that an order and technique for placement (donning) and removal (doffing) of personal protective equipment be strictly adhered to (ideally using a mirror or surveillance by another health professional), ensuring proper sealing of the face mask, with additional care during the removal procedure to avoid contamination of oneself, others and the environment.
It **is recommended** that all health professionals involved in the provision of clinical care have training and practical experience in the procedures for donning and doffing personal protective equipment prior to contact with patients.

**Table:**

**Organization of services**
**It is recommended** that the management of all level 2 (intermediate) and 3 (intensive) patients in the hospital (regardless of the service in which they are located) be performed by intensive care unit specialists in strict coordination with the Clinical Management, Directorate-General of Health and Ministry of Health.
**It is recommended** that in hospitals where there is more than one intensive care unit, a cohort area of confirmed critical cases of COVID-19 be created and a cohort area of suspected critically ill patients (for transient hospitalization) be considered, namely, establishing criteria for activation.
Isolation in a single room with negative pressure, a shower, private bathroom and adequate ventilation system, with capacity for at least 6-12 air changes/hour, **is recommended**. Once these resources are exhausted, it **is recommended** that patients be isolated in a single room with a ventilation system capable of at least 6-12 air changes/hour. When individual isolation rooms are not available, isolation in a cohort **is recommended**, respecting a minimum distance greater than 1 m between patients.
The delimitation of risk areas and predefined routes for professionals, patients and waste **is recommended.**
**It is recommended to** restrict visitations to all patients and limit the number of professionals in contact with patients (ideally with dedicated professionals), with the implementation of alternative, remote ways of communication between patients and families and between clinical teams, patients and families, regardless of the place of isolation.
**Oxygen therapy, respiratory support and adjuvant therapies**
In patients with COVID-19, it **is recommended** to administer conventional oxygen therapy (through a nasal cannula or a Venturi mask) if peripheral oxygen saturation (SpO_2_) < 90%, with the goal of an SpO_2_ between 92% and 96%.
When using nasal cannulae, it **is suggested** to place a surgical mask over the oxygen supply device.
When using a Venturi mask, a device that incorporates a filtering medium in the exhalation ports or, optionally, the placement of a surgical mask under the oxygen supply device **is suggested**.
It **is suggested**, in patients with COVID-19, in the failure of conventional oxygen therapy (peripheral oxygen saturation-SpO_2_ < 92% with fraction of inspired oxygen (FiO_2_) > 0.6, increased respiratory work and/or respiratory rate ≥ 30 cpm) consider, in the absence of criteria for endotracheal intubation, a trial of non-invasive ventilatory therapies (high-flow nasal cannulae (HFNC) or noninvasive mechanical ventilation (NIV)) provided that (1) professionals use contact, droplet and airway precautions (ideally in rooms or areas with negative pressure) and strategies aimed at minimizing aerosol production are used; (2) a protocol suitable for respiratory failure is established and implemented; (3) the technique is initiated in a highly monitored environment to avoid delays in endotracheal intubation in the event of failure of response; and (4) failure criteria are established and respected.
It **is suggested** that the choice between noninvasive ventilatory therapies (HFNC and NIV)) is based on weigh individual risks and benefits as well as on the availability of equipment/interfaces and local experience of the staff.
It **is suggested** that if a decision to use high-flow oxygen therapy via nasal cannula is made (1) a surgical mask should be placed over the nasal cannulae; (2) nasal cannulas should be adapted to the size of the nostrils, with a flow rate of 50 - 60L/minute and FiO_2_ titrated for SpO_2_ between 92% and 96%; (3) the ROX index should be evaluated at 2, 6 and 12 hours, with maintenance of support if ≥ 4.88, in the absence of criteria for endotracheal intubation; and (4) in case of failure, treatment should be optimized, considering increased support up to 60L/minute in a prone position, a transition to NIV or endotracheal intubation (and invasive ventilatory support).
It **is suggested** that if noninvasive mechanical ventilation is initiated, (1) interfaces with maximum sealing should be used, as well as specific ventilators and ventilatory circuits with antibacterial/antiviral filters; (2) ideally, non-invasive ventilation (NIV) helmets or, optionally, face masks (or oronasal) capable of specific configurations for continuous positive airway pressure (CPAP; up to a maximum of 12 - 14cmH_2_O) or bilevel positive airway pressure (BPAP; with support pressure to maintain tidal volume between 6 and 8mL/kg), FiO_2_ titrated to SpO_2_ between 92% and 96% should be used; (3) PaO_2_/FiO_2_ should be evaluated at 1 hour with maintenance of support and improvement (ΔPaO_2_/FiO_2_) ≥ 30%, in the absence of criteria for endotracheal intubation; and (4) in case of failure, therapy should be optimized, considering increased support in a prone position, eventual transition to HFNC therapy in a prone position or endotracheal intubation (and invasive ventilatory support).
A structured prone protocol (when awake) **is suggested** for all patients under HFNC therapy or NIV able to comply with orders, as long as clinically tolerated.
A structured protocol for weaning from noninvasive ventilatory therapy **is suggested**.
It **is suggested** that the decision of endotracheal intubation be based on a composite evaluation of the oxygenation state (as assessed by the ROX index and/or PaO_2_/FiO_2_) and ventilation (respiratory acidosis with pH < 7.30) but also on the respiratory effort perceived by the patient.
We **suggest** a structured protocol for endotracheal intubation, performed by an experienced operator, using contact, droplet and airway precautions (ideally in a negative pressure room).
It **is suggested** that after intubation and invasive ventilatory support, the following be used: (1) a classic ventilation strategy based on the ARDS Network protocol (tidal volume of 4 - 6mL/kg of ideal body weight with an upper limit plateau pressure < 30cmH_2_O) with minimum respiratory rate for pH > 7.30 associated with a driving pressure < 15cmH_2_O; (2) ventral decubitus for minimum periods of 16 hours if PaO_2_/FiO_2_ < 150mmHg; (3) neuromuscular blockers for ≤ 48 hours if PaO_2_/FiO_2_ < 150mmHg or severe dyssynchrony or elevated respiratory drive not controlled by optimized analgesics; and (4) in mild ARDS (PaO_2_/FiO_2_ between 200 - 300mmHg), the use of low PEEP, and in moderate to severe ARDS (PaO_2_/FiO_2_ < 200mmHg), application of high PEEP only after an evaluation of recruitment potential.
It **is recommended** that routine use of inhaled nitric oxide is not used. A structured protocol for weaning and extubation of invasive ventilatory support **is suggested**.
It **is suggested** to consider tracheotomy from the 10th day of mechanical ventilation.

**Table:**

**Bronchofibroscopy and inhalation therapy**
It **is suggested** to reserve bronchofibroscopy for urgent situations (for example, atelectasis with ventilatory impairment and critical obstruction of the central airway) or when the examination results may lead to a significant modification in the therapeutic strategy (for example, suspicion of coinfection or superinfection).
It **is suggested** that if a decision is made to perform bronchofibroscopy, the technique should be performed by the most experienced operator, and airway precautions should be used (with, ideally, the procedure occurring in a negative pressure room).
Disposable video bronchoscopes and the operator to the rear of the patient’s head **are suggested**.
It **is suggested** that when the administration of inhalation therapy is clinically indicated, pneumatic, ultrasonic or oscillatory membrane nebulization systems should not be used.

**Table:**

**Extracorporeal life support**
It **is recommended** that critically ill patients with respiratory failure associated with COVID-19 be referred for extracorporeal respiratory support after optimized invasive mechanical ventilation and associated adjuvant strategies fail.
It **is recommended** that critically ill patients with cardiogenic shock associated with COVID-19 be referred for extracorporeal cardiorespiratory support when conventional therapy fails.
It **is recommended** that the referral of critically ill patients with respiratory failure and/or cardiogenic shock associated with COVID-19 and indications for extracorporeal life support be restricted to reference centers recognized by the Ministry of Health and the General Directorate of Health.
It **is recommended** that the interhospital transfer of critically ill patients with respiratory failure and/or cardiogenic shock associated with COVID-19 and indications for extracorporeal life support occur within the reference center and be conducted, whenever possible, by an *in loco* dedicated rescue team.

**Table:**

**Other organ support**
A conservative fluid therapy strategy **is recommended** for critically ill patients with COVID-19, especially in the absence of shock.
It **is recommended** that septic shock in critically ill patients with COVID-19 be treated based on the clinical guidelines applicable to patients with septic shock not associated with COVID-19.
It **is recommended** that nonpulmonary organ dysfunction in critically ill patients with COVID-19 be managed based on the clinical guidelines applicable to non-COVID-19 patients.

**Table:**

**Coinfection, superinfection and antimicrobials**
In critically ill patients with suspected severe pneumonia combined with seasonal influenza, it **is recommended** to start antibiotic therapy for influenza and reassess the clinical picture after obtaining cultural and laboratory results.
In critically ill patients with **COVID**-19, in the presence of septic shock, it **is recommended** to administer antibiotic therapy until obtaining cultural results that allow the affirmation or exclusion of the coexistence of bacterial infection.
It **is recommended** to reassess decisions regarding antibiotic therapy initiated at admission up to 72 hours, depending on the microbiological results available, the clinical evolution and inflammatory biomarkers (namely, procalcitonin).
For critically ill patients with COVID-19, it **is recommended** to maintain a high index of suspicion for nosocomial infection (namely, ventilator-associated pneumonia).
In critical patients with COVID-19 without a microbiological diagnosis or with unfavorable progression under appropriate antibiotic therapy, it **is suggested** to consider invasive pulmonary aspergillosis associated with COVID-19.

**Table:**

**Antiviral drugs**
In critically ill patients infected with SARS-CoV-2 who require a noninvasive ventilatory strategy (NIV or HFNC therapy), invasive ventilatory support, extracorporeal respiratory support or vasopressors **are recommended**; remdesivir **is not recommended**.
In critical patients infected with SARS-CoV-2 who require conventional oxygen therapy, it **is suggested** to consider the use of remdesivir in the first 72 hours after the first positive SARS-CoV-2 test.
It **is suggested** that in patients infected with SARS-CoV-2 previously treated with remdesivir with clinical deterioration, requiring escalation of ventilatory support and corticosteroid therapy, remdesivir should be maintained until the completion of the therapeutic course.
In critically ill patients infected with SARS-CoV-2, the nonroutine use of other antivirals outside the scope of clinical use protocols or clinical trials **is recommended**.

**Table:**

**Corticosteroids and immunomodulators**
It **is recommended** that patients with COVID-19 who do not require oxygen therapy or ventilatory support should not be treated with corticosteroids unless indicated for other reasons (for example, previous therapy, acute asthma, exacerbation of chronic obstructive pulmonary disease or septic shock without response to vasopressors).
It **is recommended** that patients with COVID-19 who require oxygen therapy or ventilatory support (invasive mechanical ventilation, noninvasive mechanical ventilation or high-flow oxygen therapy by nasal cannula with a flow greater than 30 L/minute and FiO_2_ > 0.40) and are beyond 7 days since the onset of symptoms should be treated with dexamethasone 6 mg per day intravenously or enterically for up to 10 days.
It **is suggested** that for previous indications, if dexamethasone is not available, hydrocortisone (50 mg every 6 hours, intravenously), methylprednisolone (32mg daily, intravenously) or prednisolone (40 mg daily, intravenously or enterally) should be administered.
It **is suggested** that patients with COVID-19 with C-reactive protein ≥ 7.5mg/dL, ventilatory support (invasive mechanical ventilation, noninvasive mechanical ventilation or high-flow oxygen therapy by nasal cannula with a flow greater than 30L/minute and FiO_2_ > 0.40) and clinical deterioration (escalation of ventilatory support and/or worsening of PaO_2_/FiO_2_), despite corticosteroid therapy, should be treated with 8mg/kg tocilizumab (up to a maximum of 800mg) intravenously (taken only) in the first 24 hours after the start of support (must be < 14 days of hospitalization), once contraindications and other causes of deterioration of respiratory failure are excluded (for example, bacterial infection, pulmonary thromboembolism, and heart failure).
It **is suggested** that in for previous indications, if tocilizumab is not available, sarilumab (400mg) should be administered intravenously (single dose).
It **is suggested** that patients with COVID-19 receiving ventilatory support (invasive mechanical ventilation, noninvasive mechanical ventilation or high-flow oxygen therapy by nasal cannula with a flow greater than 30 L/minute and FiO_2_ > 0.40) with moderate to severe ARDS (PaO_2_/FiO_2_ < 200) and contraindications for tocilizumab should be considered for other corticotherapy protocols.
In critically ill patients infected with SARS-CoV-2, it **is recommended** *not* to use other immunomodulators outside the scope of clinical use protocols or clinical trials.
**Anticoagulation**
It **is recommended** that critically ill patients with COVID-19 with confirmation (or high clinical suspicion) of thromboembolic disease receive therapeutic strategies, including reperfusion (pharmacological and/or mechanical) and/or therapeutic anticoagulation regimens following standard institutional protocols.
It **is recommended** that critically ill patients with COVID-19, previously under a therapeutic anticoagulation regimen at home, maintain a therapeutic anticoagulation regimen. A transition from parenteral anticoagulant agents (for example, low molecular weight heparin or unfractionated heparin) to oral anticoagulants (for example, dicoumarin or new oral anticoagulants) is suggested.
It **is recommended** that critically ill patients with COVID-19 without evidence of thromboembolic disease should be medicated with a prophylactic anticoagulation regimen (standard or adjusted) in the absence of contraindications.
It **is recommended** that critically ill patients with COVID-19 receiving extracorporeal organ support (including veno-venous or veno-arterial extracorporeal life support and renal support therapy) receive antithrombotic therapy following standard institutional protocols.

**Table:**

**Other therapies**
In critically ill patients infected with SARS-CoV-2, it **is recommended** *not* to use convalescent plasma therapy outside the scope of clinical use protocols or clinical trials.
In critical patients infected with SARS-CoV-2, it **is recommended** *not* to use therapy with mesenchymal stem cells outside the scope of clinical use protocols or clinical trials.
In critically ill patients infected with SARS-CoV-2, it **is recommended** *not* to use therapy with neutralizing antibodies outside the scope of clinical use protocols or clinical trials.
In critically ill patients infected with SARS-CoV-2, routine nonsuspension of chronic therapy with renin-angiotensin system inhibitors (angiotensin converting enzyme (ACE) inhibitors or angiotensin 2 receptor antagonists) or statins **is recommended**.
In critically ill patients infected with SARS-CoV-2, it **is recommended** not to discontinue or avoid treatment with nonsteroidal antiinflammatory drugs (NSAIDs) when clinically indicated.

**Table:**

**Criteria for cure and suspension of isolation**
It **is recommended** that obtaining a cure criterion (and consequent suspension of isolation) of patients with severe or critical COVID-19 (or severe immunosuppression, regardless of the severity of the disease) does not depend on laboratory criteria but rather on the cumulative fulfillment of criteria: (1) clinical (significant improvement of symptoms with apyrexia, without use of antipyretics, for three consecutive days) and (2) temporal (20 days since the onset of symptoms).
